# Highly Durable, Stretchable Multielectrode Array for Electro-mechanical Co-stimulation of Cells

**DOI:** 10.34133/bmr.0030

**Published:** 2024-06-30

**Authors:** A Ri Kim, Sajal Shrivastava, Han-Byeol Lee, Nae-Eung Lee

**Affiliations:** ^1^ Department of Nano Science and Technology, Sungkyunkwan University, Suwon, Gyeonggi-do 16419, Republic of Korea.; ^2^ Department of Radiology, University of Pittsburgh, Pittsburgh, PA 15213, USA.; ^3^ School of Advanced Materials Science & Engineering, Sungkyunkwan University, Suwon, Gyeonggi-do 16419, Republic of Korea.; ^4^ Advanced Institute of Nano Technology, Sungkyunkwan University, Suwon, Gyeonggi-do 16419, Republic of Korea.; ^5^ Samsung Advanced Institute for Health Sciences & Technology, Sungkyunkwan University, Suwon, Gyeonggi-do 16419, Republic of Korea.

## Abstract

Electro-mechanical co-stimulation of cells can be a useful cue for tissue engineering. However, reliable co-stimulation platforms still have limitations due to low durability of the components and difficulty in optimizing the stimulation parameters. Although various electro-mechanical co-simulation systems have been explored, integrating materials and components with high durability is still limited. To tackle this problem, we designed an electro-mechanical co-stimulation system that facilitates uniaxial cyclic stretching, electrical stimulation, and optical monitoring. This system utilizes a robust and autoclavable stretchable multielectrode array housed within a compact mini-incubator. To illustrate its effectiveness, we conducted experiments that highlighted how electro-mechanical co-stimulation using this system can enhance the maturation of cardiomyocytes derived from human induced pluripotent stem cells. The results showed great potential of our co-stimulation platform as an effective tool for tissue engineering.

## Introduction

Mechanical and electrical stimulations have been used for tissue engineering using various cells. Mechanical stimulation involves subjecting the cells or tissues to mechanical forces, such as stretching or compression, to mimic the natural mechanical environment experienced by native tissues that can enhance cell proliferation, differentiation, alignment, and physiology [1–8]. On the other hand, electrical stimulation utilizes electric fields to influence cell viability, proliferation, differentiation, and signaling behavior [9–13]. Based on the benefits of 2 stimulation cues for tissue engineering, the co-stimulation of both mechanical and electrical stimuli holds great potential for tissue engineering as it can synergistically enhance tissue regeneration [3,7,9,14–16]. However, there are still challenges in identifying the optimal combinations of mechanical and electrical stimuli and developing an integrated co-stimulation system capable of long-term co-stimulation.

Electro-mechanical co-stimulation of cardiac [3,7,9,14–16], bone [17], skeletal muscle [18,19], nerve [20], and cartilage [21] tissues has been investigated for engineering of tissue constructs. Among those, electro-mechanical co-stimulation for cardiac tissue engineering is of great interest because it can enhance the maturation of tissue organization, functionality, and the expression of cardiac-specific markers [3,7,9,14–16]. Though there have been many developments in stem cell-based approaches to construct cardiac tissues (for example, using reprogrammed human induced pluripotent stem cells (hiPSCs) that can be differentiated into a variety of cell lines including cardiomyocytes (CMs) [22–25]), there have been difficulties in fully replicating the electro-mechanical function of the myocardium. To overcome this issue, controlling the microenvironment of cell culture and differentiation for the maturation of hiPSC-derived CMs (hiPSC-CMs) is known to be a critical parameter that can improve the effectiveness of cardiac regenerative therapy [26–34]. For this purpose, various attempts of employing active and passive devices for electrical and mechanical stimulations have been investigated to induce functional maturity in hiPSC-CMs. Compared to the application of mechanical cues (including scaffolds with varying material stiffness, compression, flow-driven shear stress, mechanical stretching, and 3-dimensional (3D) nanotopography [1–7]) or electrical cues to induce excitation–contraction coupling and synchronous spontaneous beating in CMs [9–12], mechanical and electrical co-stimulation for hiPSC-CMs has shown some promising outcomes [3,7,9,15,16]. However, investigation of long-term effects of stimulation and different combination of co-stimulation parameters is still lacking. Furthermore, there are still several challenges in achieving reproducibility and hardware durability for long-term stimulation. These issues primarily arise from the difficulty in creating stable components for co-stimulation. Therefore, the development of a durable platform that can be used for reliable long-term culture with durability for long-term co-stimulation and independent control capability of co-stimulation parameters is essential.

Herein, we propose an electro-mechanical co-stimulation platform based on a highly durable, stretchable multielectrode array (SMEA) that can simultaneously co-stimulate the hiPSC-CMs with electrical and mechanical cues. The SMEAs created on a 3D micropatterned stretchable polydimethylsiloxane (PDMS) substrate for the electrode region and electrical tracks exhibit exceptional durability when subjected to cyclic mechanical stretching, electrical pulsing, and autoclaving in a liquid environment. The SMEA device with a small culture well is integrated within a mini-incubator, and it provides the functions of gas supply, humidity and temperature control, electrical pulsing, and uniaxial stretching control during co-stimulation. The SMEA device is loaded into an automatic cell incubator during proliferation and differentiation stages of hiPSCs and in the interim during co-stimulation experiments. The integration of all the components in a mini-incubator allows a simple structure with a small footprint for co-stimulation. The capability to independently control a wide range of electrical and mechanical stimulation conditions has empowered us to regulate the maturation level of hiPSC-CMs. The maturation of hiPSC-CMs was more effective when co-stimulations were applied, as opposed to subjecting them to either mechanical or electrical stimulation alone. The morphology and functional characteristics of the hiPSC-CMs within the SMEA device could be assessed using optical and fluorescence microscopy. The proposed approach holds substantial promise for tissue engineering, offering enhanced maturity that can be applied in various domains of applications.

## Materials and Methods

### Fabrication of stretchable 3D micropatterned substrate

To produce a stretchable 3D micropatterned PDMS, a positive photoresist (ma-P 1275HV, Micro Resist Technology) is first patterned on a glass substrate (Taewon Scientific) by a double photolithography method. Then, a PDMS (Sylgard A+B, 10:1, Dow Corning) and a poly(urethane acryl) (PUA) mold are produced in order by a soft lithography method [35]. Then, the PDMS mixture was poured into the PUA master mold to a thickness of about 500 μm, and it was then cured for 2 h naturally for even flatness in an oven at 80 °C. The stretchable 3D micropatterned PDMS substrate was removed from the master mold, cut into 1.5-cm × 3.5-cm sections, and prepared as a substrate.

### Fabrication of the SMEA device with a cell culture well

After attaching a stencil mask manufactured to match the shape of the electrode to the 3D micropatterned PDMS substrate, a 3-nm-thick Al_2_O_3_ adhesion promotion layer was deposited by atomic layer deposition. Then, e-beam evaporation was used to deposit a 7-nm-thick Ti glue layer and a 63-nm-thick Au electrode layer using a shadow mask for formation of electrode array. Six holes for the opening of the electrode tip areas were punched into the encapsulation layer with a diameter of 350-μm-thick PDMS using a microscale punching needle to enable electrical signal transmission. The encapsulation layer and 3D micropatterned PDMS substrate were aligned and treated with oxygen plasma to create strong Si–O–Si bonds between the 2 layers. Oxygen plasma (O_2_ flow rate of 1,500 sccm and N_2_ flow rate of 500 sccm) in a microwave plasma reactor was applied for 20 s for this purpose. For strong adhesion of the 2 layers, the assembly was kept under a pressure of 600 psi in a hot-pressing machine at 90 °C. For the cell culture, a cylindrically shaped PDMS well with an opening was attached to the top of the encapsulation layer. For fabrication of PDMS well, PDMS mixture was poured into a 48-well plate (Corning), a 10-ml serological tube was inserted to close the hole, and then it was cured in an 80 °C oven for 2 h. After washing sufficiently with ethanol, the PDMS was subjected to oxygen plasma treatment (O_2_ flow rate of 1,500 sccm and N_2_ flow rate of 500 sccm) for 30 s to create a hydrophilic surface before using the device.

### Evaluation of SMEA device

Device stability and durability before and after mechanical and electrical stimulation in electrolytic condition were evaluated using electrical impedance measurements after various stimulations. The morphological change of the SMEAs were evaluated using field-emission scanning electron microscopy (FE-SEM, JEO JSM-6500F) and optical microscopy (TH3, Olympus). To assess the autoclaving feasibility of the SMEA, the devices that had undergone stimulations in an electrolytic environment were subjected to autoclaving at an elevated temperature of 121 °C and a high pressure of 26 PSI for sterilization.

### Setup of the electro-mechanical co-stimulation platform

Inside a commercial mini-incubator (Live Cell Instruments) with temperature control, medium supply and O_2_/CO_2_ gas supply functions, a customized printed circuit board (PCB) capable of applying electrical stimulation from the electrodes was installed, and a motor with cyclic uniaxial extension was installed on the right side of the incubator. This modified system allows to provide mechanical and electrical stimuli independently at desired values. The system minimizes cell damage during stimulation while maintaining suitable conditions for extended culture and monitoring, including precise temperature, humidity, and carbon dioxide levels.

### Seeding, proliferation, and differentiation of hiPSCs on the SMEA

The hiPSCs used in the experiment were cells from cord blood and a cell line purchased from 2 providers (Thermo Fisher Scientific and Accegen). They were cultured on 100-mm Petri dishes coated with recombinant human protein vitronectin (VTN-N) truncated recombinant protein (Gibco, Thermo Fisher Scientific). The hiPSCs obtained from Thermo Fisher Scientific were maintained in Essential 8 Medium (Gibco, Thermo Fisher Scientific), and the hiPSCs obtained from Accegen were maintained with mTeSR (STEMCELL Technologies) at 37 °C with 95% air and 5% CO_2_. The hiPSCs were subcultured in the devices at 70% to 80% confluence using TrypLE Express Enzyme (Gibco, Thermo Fisher Scientific). First, we coated the culture wells with 150 μl of VTN-N mixed with Dulbecco’s phosphate-buffered saline (DPBS) solution at a concentration of 5 μg/ml and incubated them at room temperature for 1 h. After removing the VTN-N at d 1, hiPSCs were seeded on the devices at a confluency of 1 × 10^5^/ml (total of 4 × 10^4^ cells in the culture well).

The hiPSC medium was changed every day for 3 d, and the seeded hiPSCs were allowed to proliferate until a confluency of 80% was reached. The seeded hiPSCs were then differentiated using a differentiation kit (PSC CM differentiation kit, Gibco, Thermo Fisher Scientific), which comprises 3 components: differentiation medium A, differentiation medium B, and maintenance medium. The detailed composition and mechanisms of the solutions are not disclosed by the supplier.

• Cardiomyocyte differentiation medium A: Pushes hiPSCs toward mesodermal commitment via bone morphogenic protein/activin pathway activation and glycogen kinase 3 inhibition
• Cardiomyocyte differentiation medium B: Induces cardiac mesoderm via Wnt inhibition
• Cardiomyocyte maintenance medium: Matures cardiomyocytes

It was informed from the supplier that hiPSC-CMs differentiated using the kit become more ventricular over time. It was also suggested that Wnt signaling modulation of hiPSCs leads to predominant differentiation toward the ventricular lineage [36].

After removing the hiPSC culture medium at d 3, we added CM differentiation medium A and incubated them for 2 d and replaced it with CM differentiation medium B solution at d 5. From d 7, the medium was changed to CM maintenance medium once every 2 d. From d 3 to d 12, the hiPSCs were differentiated into hiPSC-CMs. Images of hiPSCs proliferated on the devices were taken by an inverted optical microscope (AE31, MOTIC).

### Electro-mechanical co-stimulation of hiPSC-CMs

**Fig. 1.**
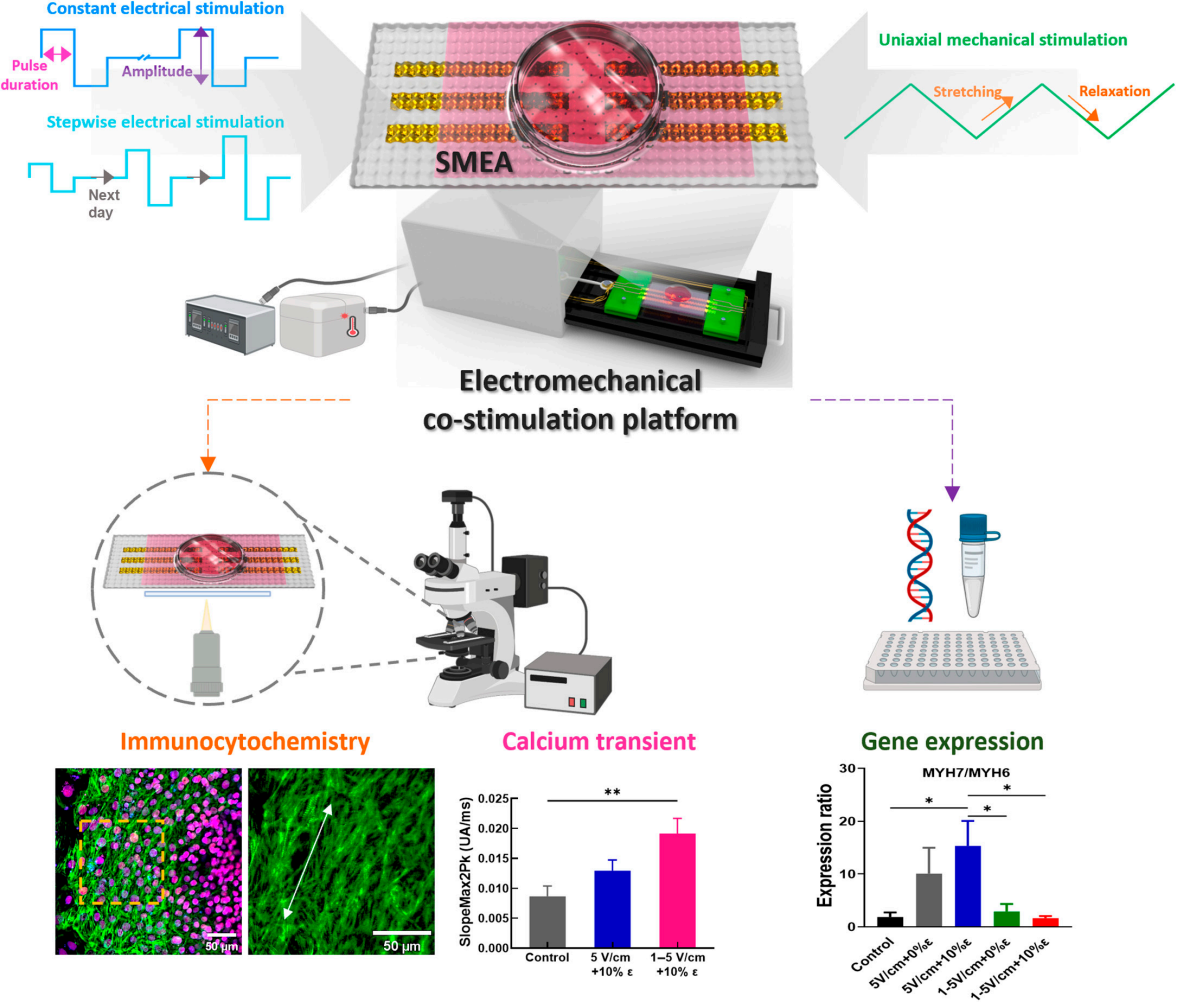
Concept of electro-mechanical co-stimulation platform. Schematic of the electro-mechanical co-stimulation system integrated with a SMEA device inside a mini-incubator for culture, co-stimulation of electrical pulsing (*E*) and uniaxial extensional straining (ε), and observation of stimulated cells. The cells can be analyzed though ICC as an end-point cell analysis (i.e., calcium transient, membrane potential, and gene expression analysis). After stimulation, the stimulated cells were harvested and used for further gene expression analysis.

The stimuli of uniaxial cyclic stretching at 1 Hz and a biphasic electrical stimulation with a duration of 1 ms were given every day for 1 h for a total of 5 d for co-stimulation of hiPSC-CMs in the SMEA device through the PCB inside the mini-incubator. The silver-colored box depicted in Fig. S1 represents the motor box of the mechanical stimulation platform. The electrical and mechanical stimulations were administered separately. The stretching speed and distance can be adjusted using the controller on the front face of the box. The distance between the fixation devices on both sides of the SMEA determines the tensile strength (%). The actual stretching process is demonstrated in Movie S1. The PCB connected to a function generator was used to deliver waveforms of the same frequency as depicted in Fig. 1, transmitting them through the cells within the SMEA. A biphasic waveform generated from a function generator (AFG3102, Tektronix) was applied. The applied electrical stimulation was transmitted to the electrodes across the opposite sides. The electrical stimulation signal was monitored through an electrometer (SMU 2612, Keithley Instruments). This approach offers the advantage of administering electrical stimulation in various waveforms, frequencies, and intensities, independently from mechanical stimulation. Thus, electrical and mechanical stimulations are independent and constitute a system capable of delivering dual stimuli across a diverse and wide range.

### Immunocytochemistry analyses of hiPSC-CMs

For immunocytochemistry (ICC) analysis of hiPSC-CMs, 200 μl of fixative solution (4% formaldehyde in DPBS, Life Technologies) was added after removing the CM maintenance medium and incubated for 15 min at room temperature. After removing the fixative solution, 200 μl of permeabilization solution (1% saponin in DPBS, Life Technologies) was added. After 15 min of incubation at room temperature, it was changed to 100 μl of blocking solution (3% bovine serum albumin in DPBS, Life Technologies) and incubated for 30 min at room temperature. Then, 0.1 μl of primary antibodies anti-NKX2-5 (host: rabbit) and anti-TNNT2 (host: mouse) were added directly into the culture well containing 100 μl of blocking solution and incubated for 4 h at room temperature. All liquids were removed, and the device was gently washed 3 times with a wash buffer (Life Technologies). The device was incubated for 1 h at room temperature in 0.4 μl of Alexa Fluor 594 donkey anti-rabbit (for anti-NKX2-5, Life Technologies) and Alexa Fluor 488 donkey anti-mouse (for anti-TNNT2, Life Technologies) secondary antibodies diluted with 100 μl of blocking solution. Then, the device was gently washed 3 times with a wash buffer. One drop of 4′,6-diamidino-2-phenylindole (NucBlue Fixed Cell Stain, Life Technologies) in 5 ml of the wash buffer for staining was added and incubated for 5 min. Imaging of ICC was carried out using a confocal microscope (FV3000, Olympus).

### Gene expression analysis of stimulated hiPSC-CMs

Ribonucleic acid (RNA) extraction. Total RNA was extracted from hiPSC-CMs using TRIzol reagent (Invitrogen) before and after simulations. First, the hiPSC-CMs were homogenized with TRIzol reagent, and 0.2 ml of chloroform was added to each tube. After vigorously shaking the tube by hand for 15 s, the tube was left to stand at room temperature for 3 min. For phase separation, the samples were centrifuged at 13,000 rpm for 15 min at 4 °C, and the upper aqueous layer was transferred to a new tube. An equal volume of isopropyl alcohol was added and incubated for 10 min at room temperature. To precipitate the RNA, the tube was centrifuged at 13,000 rpm for 10 min at 4 °C. The supernatant was removed, and the RNA pellet was washed with 1 ml of 70% ethanol. The samples were briefly vortexed and centrifuged at 13,000 rpm for 5 min at 4 °C. The supernatant was removed, and the RNA pellet was resuspended in RNase-free water.

### Measurement of yields, purity, and integrity of RNA

RNA yields from the hiPSC-CMs were measured based on the absorbance at 160 nm, and the A_260:280_ and the A_260:230_ ratios were used to assess the purity of RNA using a NanoDrop 2000 Spectrophotometer (Thermo Fisher Scientific Inc.). The RNA integrity number was determined using a high-sensitivity RNA Screen Tape Kit following the manufacturer’s protocol on an Agilent 4200 TapeStation.

### mRNA quantification by quantitative reverse transcription polymerase chain reaction

With total RNA, reverse transcription was performed using SuperScript II RNase (Invitrogen) according to the manufacturer’s instructions. The cDNA of the mRNA was amplified using the following primer pairs: MYH6 Forward: 5′-AGAGTCGGTGAAGGGCATGA-3′, Reverse: 5′-AGCCGCAGCAGGTTCTTTTT-3′; MYH7 Forward: 5′-GAGCCTCCAGAGCTTGTTGA-3′, Reverse: 5′-ACGATGGCGATGTTCTCCTT-3′; CACNA1C Forward: 5′-ATGACGAAAATCGGCAACTG-3′, Reverse: 5′-GGAAACCCCTCTTCGGAGAT-3′; ACTC1 Forward: 5′-AAGGACCTGTATGCCAACAA-3′, Reverse: 5′- CTTCTGCATACGATCAGCAA-3′; GAPDH Forward: 5′-CGAGATCCCTCCAAAATCAA-3′, Reverse: 5′-CCTTCTCCATGGTGGTGAA-3′. Real-time polymerase chain reaction (PCR) was performed on a StepOnePlus Real-Time PCR System (Applied Biosystems) using an SYBR Green PCR Kit (Applied Biosystems), according to the manufacturer’s instructions. Thermal cycling conditions were 95 °C for 10 min followed by 40 cycles of 95 °C for 15 s and 1 min at optimal *T*_m_ (59 °C). The data were analyzed using StepOne software v2.2.2 (Applied Biosystems). The expression levels of each mRNA were normalized to an endogenous control GAPDH and were calculated using the 2^−ΔΔCt^ method.

### Calcium transient assay of hiPSC-CMs

Fluorescent indicators of Ca^2+^ were used to measure calcium signaling of hiPSC-CMs stimulated by agonists and inhibited by antagonists via G protein-coupled receptors, which represent a marked and active target group in drug discovery. Calcium assay buffer (1 ml, Fluo-4 Direct calcium assay buffer, Invitrogen) was mixed with 77 mg of water-soluble probenecid (Invitrogen) to obtain a 250 mM solution of probenecid, and the mixture was agitated in a vortex until dissolved. To make a 2× calcium reagent loading solution, 200 μl of 250 mM probenecid stock solution was mixed with 10 ml of Fluo-4 Direct calcium assay buffer. The 2× calcium regent loading solution (200 μl) was added directly into the cell culture well containing 200 μl of cell culture medium. The devices were incubated at 37 °C for 30 min and at room temperature for 30 min. Confocal images of the cells on the 3D stretchable micropatterned cell culture device were taken directly. To obtain a calcium transient intensity video, the cell culture medium and calcium reagent loading solution were removed, and the cell culture well was cut off. Imaging of calcium transients was carried out using a confocal microscope (FV3000, Olympus). The calcium transient video was obtained from a fluorescence microscope (IX71, Olympus). The cell side of the devices was turned down for recording to allow a path of light at an excitation wavelength of 494 nm and an emission wavelength of 516 nm.

### Membrane potential measurements of hiPSC-CMs

Various physiological parameters (such as cell signaling and muscle contraction) of stimulated hiPSC-CMs are importantly affected by changes in the electrical potential across the membrane. To prepare a membrane-sensitive loading solution, 10 μl of membrane-sensitive dye (Fluovolt dye, Invitrogen) was mixed with 100 μl of 100× Pluronic surfactant polyols (PowerLoad Concentrate, Invitrogen). Then, 10 ml of 20 mM glucose in Hank’s balanced salt solution (HBSS) (Invitrogen, Thermo Fisher Scientific) was added and mixed. The devices containing cell culture medium were washed twice with HBSS. A membrane-sensitive loading solution was added, and hiPSC-CMs were incubated at room temperature for 30 min. The membrane-sensitive loading solution was removed, and devices were washed twice with HBSS. To take confocal images, 100 μl of 20 mM glucose stock in HBSS was added to the devices. Imaging of membrane potentials was carried out using confocal microscopy (FV3000, Olympus).

### Analysis of beating characteristics of co-stimulated hiPSC-CMs

We compared the average contraction speed from the multiple control samples and co-stimulated hi-PSC-CMs with electrical pulsing of a constant 5 V/cm and a gradual 1 to 5 V/cm at a constant cyclic stretching strain of 10%. The videos obtained from spontaneously beating hiPSC-CMs were recorded using optical microscopy (Motic AE31). Subsequently, the video files were converted into time–speed graphs using the CONTRACTIONWAVE software, which allows to calculate the contraction speed [37].

### Analysis of ICC, calcium transient, and membrane potential images

An ImageJ program (FIJI) was used for the analysis of fluorescence images including ICC, cell alignment, calcium transient, and membrane potential images. We analyzed the peaks of the calcium transient with a plug-in called Spiky in ImageJ. Statistical analysis for the calcium transient images was performed using GraphPad Prism 9, with one-way analysis of variance (ANOVA) conducted between groups. The post hoc Tukey test was used for comparisons between groups and columns. Statistically significant *P* < 0.05 in calcium transient. Data are presented as means ± standard error of the mean.

## Results and Discussion

### Design concept and fabrication of electro-mechanical co-stimulation platform

An electro-mechanical co-stimulation platform that possesses the co-stimulation capability of controlled simultaneous electrical pulsing (*E*) and mechanical strain (ε) stimulations was designed. The main components include the SMEA device housed in a mini-incubator, control electronics for co-simulations, the mini-incubator to keep the cells viable during co-stimulation, maintenance components of the culture environment, and a peristaltic pump for the supply of the culture media to the SMEA device during stimulation. The SMEA device is loaded inside the mini-incubator during stimulation and can be also loaded into a commercial culture incubator for long-term culture (Fig. 1). The SMEA device was designed in such a way that it can be mounted on an optical or confocal microscope for the observation of morphological change and functional analysis of stimulated cells or tissues. In our design, the SMEA device has 6 Au electrodes. The mini-incubator during co-stimulation experiments was tailored to maintain optimal levels of CO_2_, humidity, and temperature, thereby minimizing cell damage during stimulation. A peristaltic pump is used to supply culture media during co-stimulation. A specifically designed PCB enabled an electrical connection between control electronics and the SMEA for applying the electrical and mechanical stimuli reliably. Cyclic uniaxial stretching for mechanical stimulation is carried out using a stepping motor. During co-stimulation, the status of electrical stimulation on the stimulation platform is monitored continuously using an electrometer. For the optimal operation of the system, electrical and mechanical cues can be independently combined. Photographs of the components of the multifunctional stimulation platform and the connections between them are shown in Fig. S1. After stimulation, the cells or tissues can be investigated additionally using optical or confocal microscopy for functional analysis such as ICC, calcium transients, and membrane potential measurements.

To establish an efficient co-stimulation platform, the high durability of the SMEA device in cell culture media under continuous co-stimulation for an extended period is a crucial factor to prevent electrode damage. For this purpose, the 3D micropatterned substrate which have curvilinearly connected valleys and bumps with no planar region to mitigate the cracking of the electrode under cyclic stretching was utilized to allow uniaxial stretchability of the electrodes without marked deterioration [35,38]. The stretchable 3D micropatterned substrate was molded from at ultraviolet-curable PUA mold replicated from a master mold fabricated on a Si wafer using a double-lithography method [35]. The transparency of the PDMS with relatively low background noise allowed optical imaging. For the fabrication of gold (Au) electrodes, 3 layers of Al_2_O_3_, Ti, and Au were deposited in sequence onto the 3D micropatterned substrate and then encapsulated. The substrate consisted of an array of 6 Au electrodes, enclosed by a PDMS layer, with the exception of the electrode tip area where cells adhere (Fig. S2A). Six perforations were made in the encapsulation layer to reveal the tip area of each electrode, facilitating electrical signal transmission. The encapsulated flat section of the device, where the majority of cells were located, covered 96% of the total substrate surface area. The encapsulation layer also provide a neutral plane condition, effectively diminishing the stress exerted on the electrode tracks [39]. To create a sturdy Si–O–Si bond between the encapsulation layer and the 3D micropatterned substrate, both layers underwent microwave oxygen plasma treatment and were then hot-pressed for the bonding process (Fig. S2B) [40]. Finally, a PDMS well for the containment of cells and culture medium was attached on the substrate with microelectrodes. The picture of the fully assembled SMEA with the PDMS well attached is shown in Fig. S2C. Movie S1 shows operation of the constructed platform that provides both electrical and mechanical co-stimulation.

### Durability evaluation of SMEA device under co-stimulation in culture media

To evaluate the resilience of the SMEA device, we performed mechanical stretching and electrical pulsing tests on the SMEA in culture media. Subsequently, we examined the morphological changes in the electrodes. Additionally, we assessed the electrical stability of devices on both planar and 3D micropatterned substrates by conducting impedance measurements, and we compared the results after applying mechanical and electrical stimulations.

**Fig. 2.**
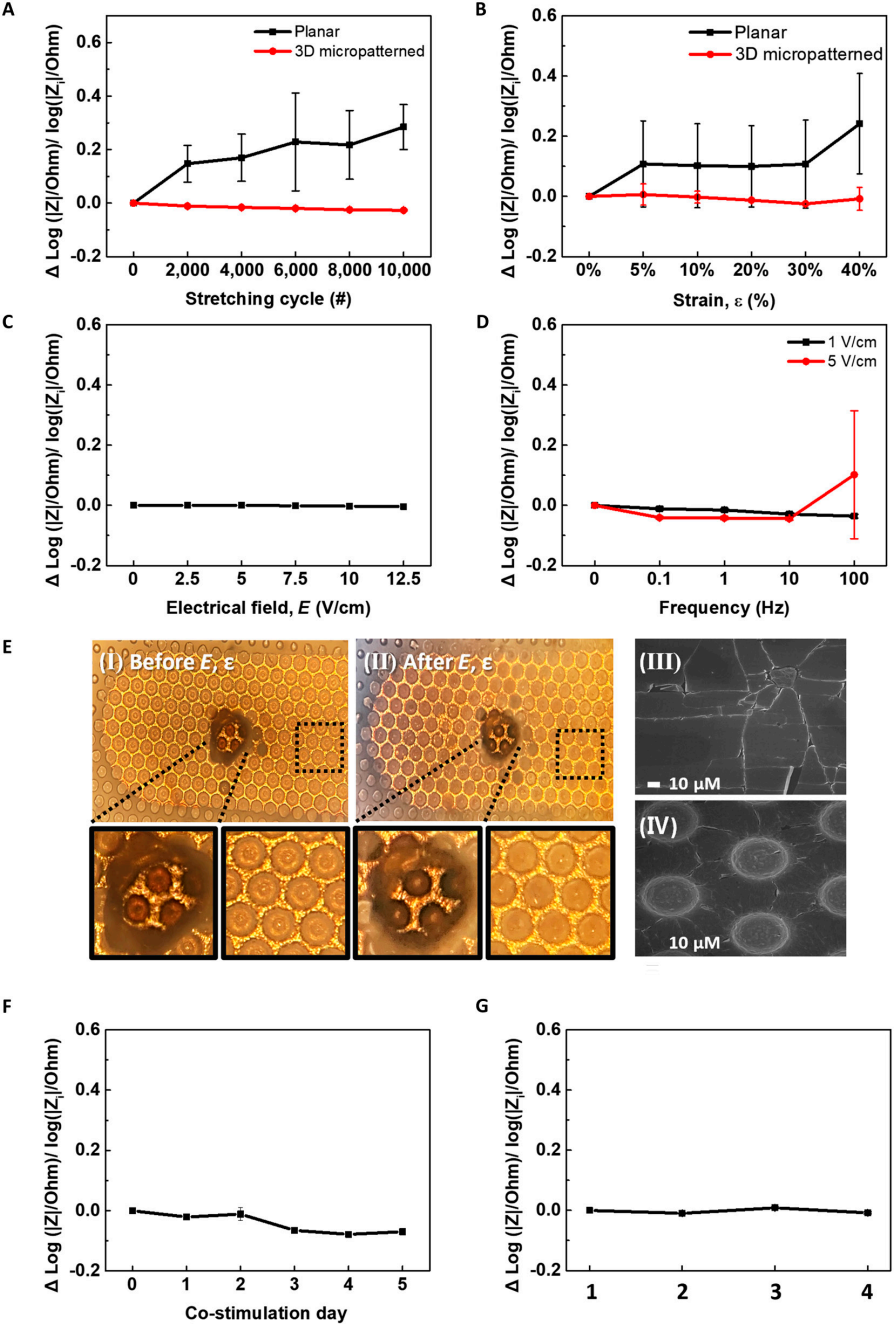
Evaluation of device durability and functionality of SMEA device. (A) Effects of the mechanical stretching cycles on the average impedance changes, Δlog|Z|/log|Z_i_|, of the SMEAs on planar and 3D micropatterned substrates at 10% ε. (B) Effects of different stretching strain (ε) on the Δlog|Z|/log|Z_i_| of the SMEAs made of planar and 3D micropatterned substrates with 10,000 stretching cycles at 0% to 10% ε. (C) The Δlog|Z|/log|Zi| response to the variation of electrical pulsing condition for an SMEA made of a 3D micropatterned substrate. (D) The Δlog|Z|/log|Zi| response to the variation of frequency of electrical stimulation at 1 and 5 V/cm for an SMEA made of a 3D micropatterned substrate. (E) Optical and FE-SEM images of the electrodes on 3D-micropatterned PDMS substrate before (I and III, respectively) and after (II and IV, respectively) co-stimulation in culture medium, respectively. (F) The Δlog|Z|/log|Z_i_| response to the co-stimulation of 1-Hz biphasic electrical field of 5V/cm and 1-Hz mechanical stimulation of 10% ε for 1 h every day for 5 d. (*n* = 4, The deviation from the mean value for each condition for d 0 ; 0 ± 0, for d 1 ; −0.01978 ± 0.002545, for d 2 ; −0.01738 ± 0.018538, for d 3 ; −0.06458 ± 0.002854, for d 4 ; −0.0786 ± 0.002981, and for d 5 ; −0.06982 ± 0.001623). (G) The Δlog|Z|/log|Z_i_| response to the co-stimulation experiment before and after autoclaving. 1: Before the first stimulation, 2: After the first stimulation, 3: After autoclaving, 4: After the second stimulation (*n* = 3, for 1; 0 ± 0, for 2; −0.00982 ± 0.00143, for 3; 0.00846 ± 0.00233, for 4; −0.00816 ± 0.00329).

First, to compare the durability of the electrodes on planar and 3D micropatterned substrates, both planar and 3D-micropatterned electrodes were subjected to an increasing number of cyclic stretches, up to 10,000 times at a fixed strain of 10%, and electrical impedance analysis was performed. The averaged impedance changes, Δlog|Z|/log|Z_i_|, where Δlog |Z| is the difference between log|Z_f_| and log|Z_i_| and the |Z_f_| and |Z_i_| are the initial impedance values before and after stimulation, respectively, were obtained from the impedance spectra from the electrodes in the SMEA devices (Figs. S3 and S4 for planar and 3D micropatterned substrate, respectively). The results indicated that the electrodes on the 3D micropatterned substrate maintained stable impedance values even after 10,000 stretching cycles at 10% ε, but those on the planar substrate resulted in a gradual increase in the impedance values (Fig. 2A), indicating deterioration of the electrodes. The exceptional stability of the SMEA array on the 3D micropatterned stretchable substrate under repetitive uniaxial mechanical stimulations in the culture medium is attributed to their unique morphological characteristics. The results suggest that the SMEA based on the 3D micropatterned substrate can be used for stable mechanical stimulations in the cell culture medium. Following the impedance measurements with cyclic stretching, the surface morphological changes of SMEAs on the planar and 3D micropatterned PDMS substrates were also examined. The FE-SEM image in Fig. S5A shows that the Au electrode on the planar substrate appears to be substantially damaged, whereas that of the Au electrode on the 3D micropatterned substrate in Fig. S5B revealed only fine cracks without significant damage. This observation is consistent with the impedance measurement results in Fig. 2A, further supporting the superior performance and robustness of the SMEAs on 3D micropatterned substrate under mechanical stimulation in a culture medium.

To further assess the durability of the SMEA device under varying levels of applied ε, we examined the electrical stability of both planar and 3D micropatterned substrate devices. This evaluation involved impedance measurements after 60 stretching cycles, during which the stretching strain was varied from 0% to 40%. The results were then compared. The averaged Δlog|Z|/log|Z_i_| values obtained from the impedance measurements of 3 electrodes in the SMEA device (Figs. S6 and S7 for planar and 3D micropatterned substrate, respectively) indicate that the devices on the 3D micropatterned substrate maintain stable impedance characteristics even at 30% ε but those on the planar substrate resulted in a sudden increase of the impedance even at 5% ε (Fig. 2B). The Δlog|Z|/log|Z_i_| value of the devices on the 3D micropatterned substrate showed only a slight increase at 40% ε. The FE-SEM image, taken after impedance measurement after the cyclic stretching, showed much larger cracks from the electrode on the planar substrate (Fig. S8A) compared to that from the electrode on the 3D micropatterned substrate (Fig. S8B), which is consistent with the results in Fig. 2B.

Subsequently, the stability of the SMEA devices under electrical stimulation was also assessed. The averaged Δlog|Z|/log|Z_i_| values obtained from the impedance measurement data from 3 Au electrodes on the 3D micropatterned substrate with the biphasic electrical pulsing at 1 Hz applied and its applied electrical potential varied, indicating that the electrodes were stable under biphasic electrical stimulation from 2.5 to 12.5 V/cm (Fig. 2C and Fig. S9). The FE-SEM image showed no substantial damage in the Au electrodes after electrical stimulation (Fig. S10). The Δlog|Z|/log|Z_i_| values were measured under a gradually increasing electrical stimulus of 1 V/cm (Fig. S11) and 5 V/cm (Fig. S12) for 1 min. At an applied potential of 1 V/cm with a gradual increase in the frequency of electrical stimulus, the impedance value remains stable without any noticeable change. However, when subjected to electrical stimulation of 5 V/cm at 100 Hz, the Δlog|Z|/log|Z_i_| value increased (Fig. 2D). In this case, slight damage and tearing off of the Au electrode were identified from FE-SEM analysis (Fig. S13). The overall stability of the SMEA device under electrical stimulation is noteworthy, as indicated by its performance during the electrical stimulation with a lower field.

The device stability was also verified under electro-mechanical co-stimulation. The morphological changes of Au electrodes formed on the planar and 3D micropatterned PDMS substrates were observed and compared after cyclically stretching the device 50,000 times at 10% ε with a frequency of 1 Hz while applying the biphasic electrical pulsing between 2 electrodes at an *E* of 5 V/cm and a frequency of 1 Hz in the culture medium. No substantial change in the impedance before and after electro-mechanical co-stimulation was observed (Fig. S13). The optical microscope images of the cyclically stretched devices on the planar substrate indicated that many cracks already exist in the electrodes even before the co-stimulation (Fig. S15, left panel), and the crack density was increased with co-stimulation (Fig. S15, right panel). In contrast, the electrodes formed on the 3D micropatterned PDMS substrate have some wrinkles but no marked cracks before the co-stimulation (Fig. 2E, I) and after co-stimulation (Fig. 2E, II). The top-view FE-SEM images of the device after co-stimulation in the culture medium showed long and large cracks from the electrode on the planar PDMS substrate (Fig. 2E, III) but a small number of short cracks on the 3D micropatterned PDMS substrate (Fig. 2E, IV). Additionally, the Δlog|Z|/log|Z_i_| value remains stable when subjected to 1-Hz mechanical stimulation of 10% ε and 1-Hz biphasic pulsing of 5 V/cm continuously for 1 h daily over the course of 5 d (Fig. 2F).

The SMEA device was also tested for the possibility of using autoclaving to check reusability. Before and after autoclaving, the devices underwent the 2-time repetition of co-stimulation at 1-Hz mechanical stimulation of 10% ε and 1-Hz biphasic electrical stimulation of 5 V/cm. After co-stimulation of the device in the culture medium, the device was rinsed with 70% ethanol and distilled water and was then autoclaved for sterilization. A comparison of the impedance data in Fig. 2G and Fig. S16 showed that the Δlog|Z|/log|Z_i_| values before and after autoclaving and following co-stimulation of the device were not substantially different. The results indicate that the SMEA device can endure high heat at high pressure during autoclaving and can therefore be reused. After completing one cycle of the culture and stimulation experiment, we reused the undamaged SMEA devices for one more cycle. The FE-SEM images taken after experiments involving co-stimulation and autoclaving revealed minimal damage from the working electrode (Fig. S17). Throughout the characterization of the SMEAs, it was confirmed that the SMEA device can withstand a wide range of stimulation environment with high durability.

### Demonstration of co-stimulation platform for enhanced maturation of hiPSC-CMs

To demonstrate the co-stimulation potential of the resilient SMEA, we seeded and cultured hiPSCs, followed by their differentiation into hiPSC-CMs. Figure S18 illustrates the overall flow of the experiment. In addition, we subjected hiPSC-CMs to various stimulation parameters and subsequently examined their impact on the functional properties of the co-stimulated cells. Specifically, we investigated the influence of different combinations of co-stimulation conditions on the maturation level of hiPSC-CMs. To conduct this study, we employed hiPSC-CMs derived from hiPSCs because hiPSCs possessing pluripotent capabilities, including the differentiation into CMs, serve as a valuable cellular resource. The hiPSCs, derived from patient samples, give rise to hiPSC-CMs, offering a specialized tool for conducting patient-specific or drug-specific research in the realm of regenerative medicine and pharmaceutical investigations. The hiPSCs were differentiated into hiPSC-CMs using a specific differentiation kit. Details on the differentiation of hiPSC-CMs from hiPSCs for this study were described in Materials and Methods.

**Fig. 3.**
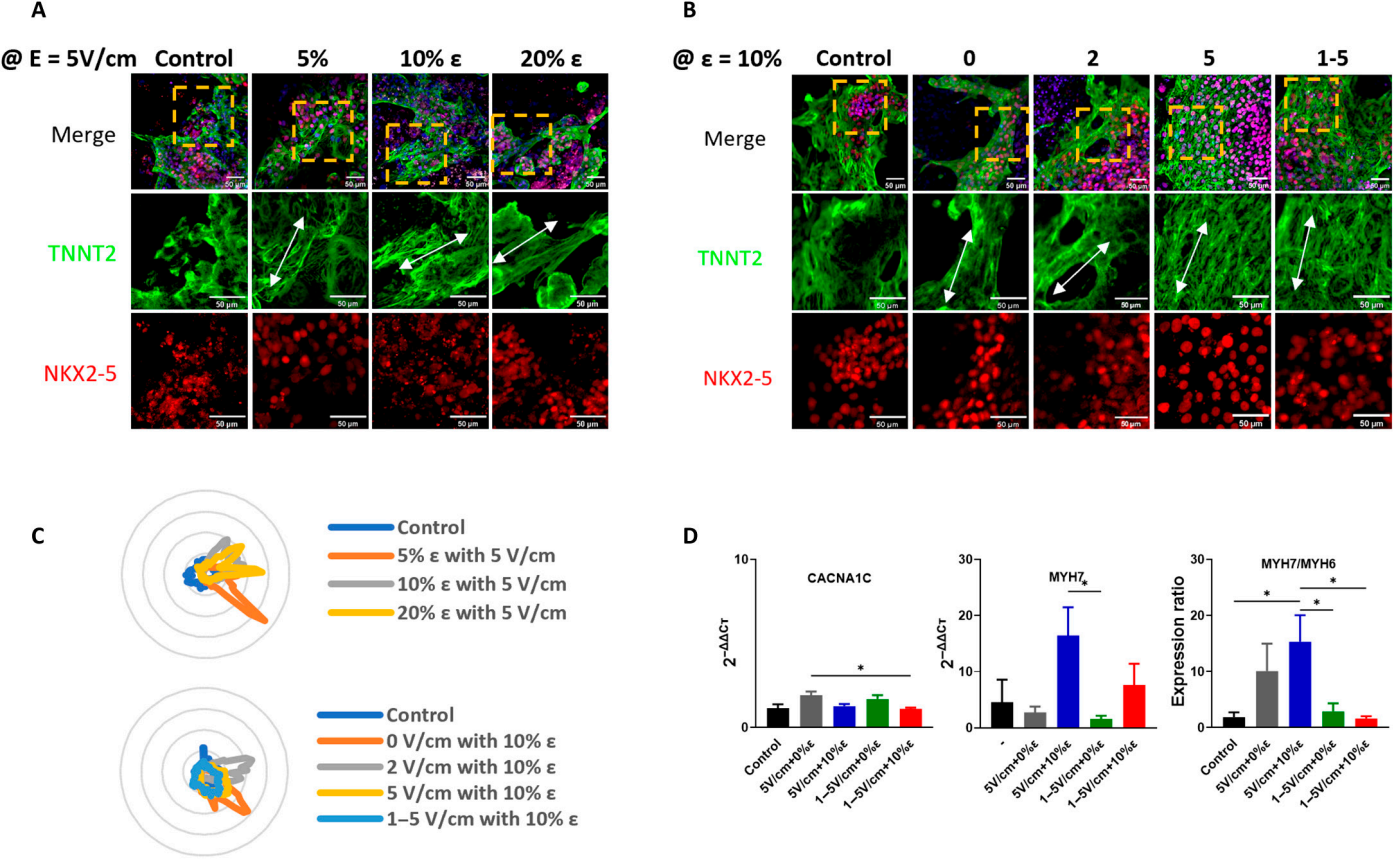
Analysis of the effects of stimulated electro-mechanical co-stimulation on maturation by immunochemistry and gene expression analysis. (A) ICC images of the samples with various ε at a constant *E* of 5 V/cm, (B) ICC images of the samples with various *E* value at a constant ε of 10%. Scale bar is 50 μm. (C) The directionality of hiPSC-CM from ICC images of the sample with various ε at a constant *E* of 5 V/cm and with various *E* values at a constant ε of 10%. (D) Gene expression analysis data of nonstimulated control hiPSC-CM sample and hiPSC-CMs samples stimulated with an *E* of 5 V/cm and ε values of 0% and 10% and gradual *E* of 1 to 5 V/cm at ε values of 0% and 10%. CACNA1C, alpha-1 subunit of a voltage-dependent calcium channel; MYH6, myosin heavy chain alpha isoform; MYH7, myosin heavy chain beta isoform. *Statistically significant differences between control, 5 V/cm with 0% ε, 5 V/cm with 10% ε, 1 to 5 V/cm with 0% ε, and 1 to 5 V/cm with 10% ε. (Error bars represent the mean value ± SEM, the sample number of control, and 5 V/cm with 0% ε for MYH7 = 2, the sample number of stimulated samples except the one with 5 V/cm at 0% ε for MYH7 = 3. Every sample was analyzed 3 times. *P* < 0.05, one-way ANOVA with the post hoc Tukey test.)

First, to evaluate the maturation characteristics of hiPSC-CMs co-stimulated at various conditions, ICC analysis of the hiPSC-CMs was carried out. For this purpose, the stimulated hiPSC-CMs were labeled with fluorescently tagged antibodies. Two key markers, NKX2-5 (which signifies the early cardiac mesoderm) and TNNT2 (which is an important marker of myocardial cells) were used. The images in Fig. 3A show the confocal microscopy images of the co-stimulated hiPSCs-CMs when the ε was varied at a constant electrical pulsing of 5-V/cm amplitude. Figure 3B represents the immunostaining results of hiPSCs-CMs stimulated at a constant ε of 10%, while the *E* value was varied. With or without applied electrical stimulation, when an ε of 10 or 20% was applied, the stimulated hiPSC-CMs were aligned with increased directionality (white arrows in Fig. 3A and B). The alignment characteristics of the hiPSC-CMs were analyzed statistically using the images of TNNT2 expression in Fig. 3A and B. It is evident from the comparison between the control and stimulated samples that there are clear differences in the directionality of the stimulated samples (Fig. 3C and Fig. S19). The images in Fig. 3C provide a quantitative directionality analysis of hiPSC-CMs based on the TNNT2 images shown in Fig. 3A and B. From the expression of TNNT2 in Fig. 3A, the fiber bundles of CMs in the samples with the ε of 5% and 10% were more prominent compared to the control sample with no stimulation. The fiber bundles of CMs in the sample with an ε of 20% were less significant compared to the sample stimulated with an ε of 5% and 10%. A stronger mechanical stimulation at a constant *E* of 5 V/cm is associated with more directional fiber bundles (Fig. 3C). However, the maturity observed from the fibrous structure of TNNT2 expression suggests that proper mechanical stimulation is necessary given the degree of subdivision in the fibrous structure. As hiPSC-CMs mature, the expression of TNNT2 increases and the proteins become more organized into a fibrous structure [30,41]. When the E was varied at the fixed ε, it was difficult to confirm the marked difference in the TNNT2 fluorescence expression intensity between the control sample and the samples with *E* values of 0 and 2 V/cm (Fig. 3B). However, the ICC images of hiPSC-CMs stimulated with an *E* of 5 V/cm and a gradual *E* of 1 to 5 V/cm at a fixed ε of 10% indicated that the expression intensity of TNNT2 is stronger and clearer at higher *E*, and thin and dense fibers are well distributed compared to the control sample (Fig. 3B).

The fluorescence expression of NKX2-5, which represents early cardiac mesoderm, did not show a clear tendency while intensity was maintained throughout the co-stimulations (Fig. 3A and B). It has been reported that the expression of NKX2-5 decreased in long-term cultures [42]. The observed expression intensity was retained, presumably because the period of the culture was not very long. Since the ICC data are qualitative, the expression data of NKX2-5 and TNNT2 were not enough to reveal all their features at every region of the sample. Thus, it was difficult to fully judge the effects of co-simulation conditions on the maturation of hiPSC-CMs with ICC analysis alone.

Therefore, to convey additional elucidation of the co-stimulation effects on maturation, gene expression analyses were also performed. The gene expression analysis was used to elucidate the effects of co-stimulation on the expression of various genes for the samples with salient features in ICC analysis (Fig. 3A and B). A compilation of the gene expression in various co-stimulated hiPSC-CMs is represented in Fig. 3D. The expression of the *CACNA1C* gene is related to the electrophysiological characteristics of action potential duration and calcium handling of storage, cycling, and dynamics. As shown in Fig. 3D, the difference in the gene expression of *CACNA1C* was not marked for the co-stimulated samples compared to the control samples, indicating that maturity in their electrophysiological properties was not clearly observed. A comparison of *MYH6* (encodes myosin heavy chain alpha [MHC-α] isoform) and *MYH7* (encodes myosin heavy chain beta [MHC-β] isoform) gene expression in samples was made under different stimulation conditions. The data in Fig. 3D show that a much higher gene expression level was observed in *MYH7* (16.44 ± 4.76 and 7.65 ± 3.55, respectively) especially when an ε of 10% was applied to the hiPSC-CMs on the SMEA devices with an *E* of 5 V/cm and gradual *E* of 1 to 5 V/cm, respectively. As hiPSC-CMs mature, the MHC-α isoform shifts to the MHC-β isoform [9,23,30,43,44]. In turn, the expression ratio of *MYH7*/*MYH6* is increased with an increase in the maturation of hiPSC-CMs [23,45]. In the expression ratio of *MYH7*/*MYH6* was plotted using the data in Fig. 3D. As shown, the *MYH7*/*MYH6* expression ratios of the stimulated hiPSC-CMs were higher than that of the control sample. The sample co-stimulated with an *E* of 5 V/cm at an ε of 10% expressed the highest *MYH7/MYH6* gene expression ratio (15.33 ± 4.46), which indicates the highest degree of maturation. The results confirmed that the co-stimulation enhanced the degree of maturation in hiPSC-CMs.

**Fig. 4.**
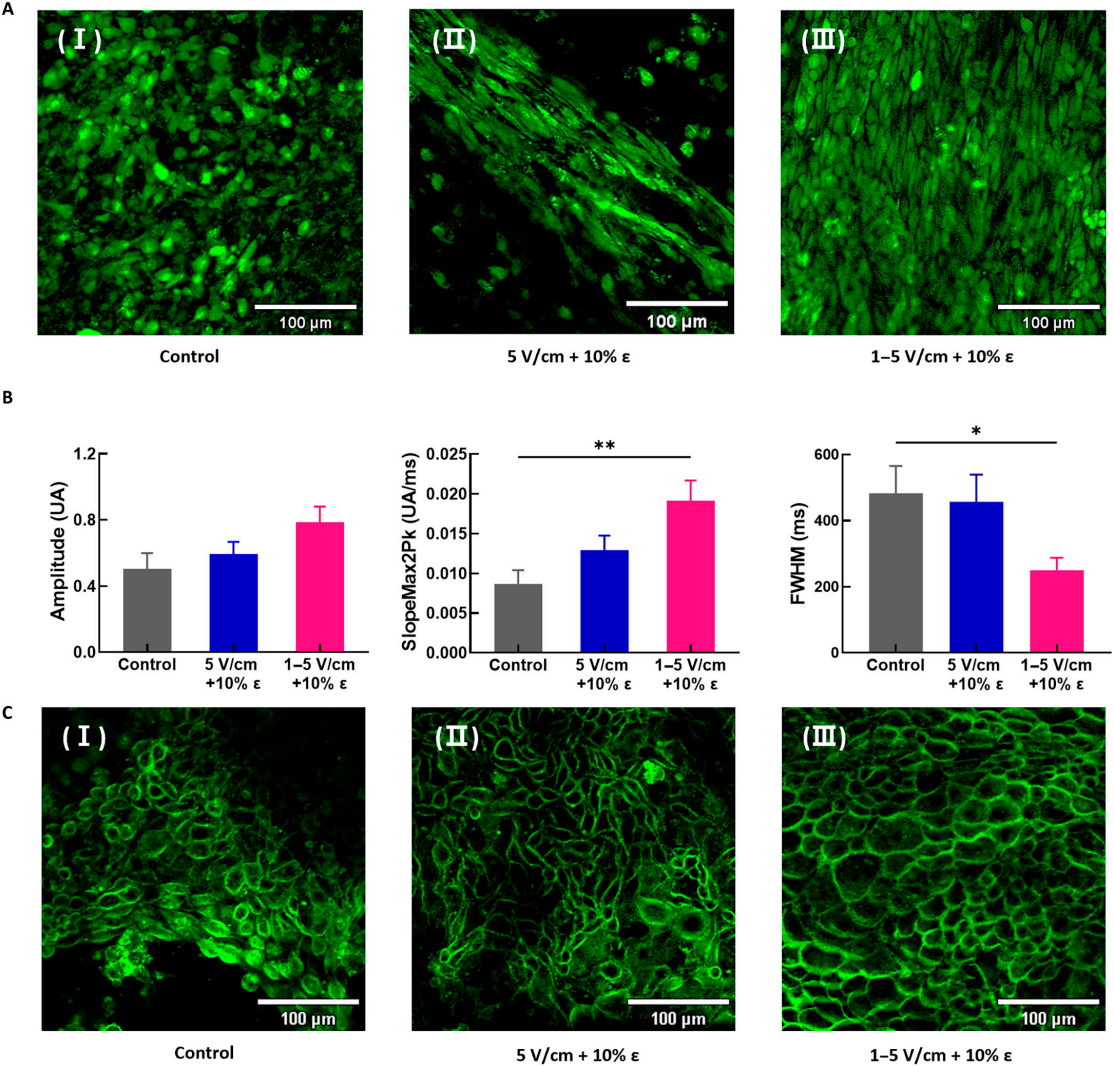
Analysis of calcium transient and membrane potential. (A) The fluorescent confocal images of calcium transients of the control hiPSC-CMs (I) and of the hiPSC-CMs co-stimulated with the *E* of 5 V/cm (II) and the gradual *E* of 1 to 5 V/cm (III) at an ε of 10%. (B) The statistical data of amplitude, absolute maximum slope from peak start to peak maximum (slopemax2Pk), and FWHM obtained from the calcium transients of hiPSC-CMs (the number of samples, *n* = 1 per group, the number of measuring points = 9 to 10 points, exposure time = 50 ms, *P < 0.05, **P < 0.005 one-way ANOVA with post hoc Tukey test). (C) Fluorescent confocal images of membrane potential of the control hiPSC-CMs (I) and of the hiPSC-CMs co-stimulated with the *E* of 5 V/cm (II) and the gradual *E* of 1 to 5 V/cm (III) at an ε of 10%.

The data depicted in Fig. 3D imply a lack of substantial variation in the gene expression of *CACNA1C*, suggesting an absence of distinct maturity in electrophysiological properties. To delve deeper into the impact of co-stimulation parameters on additional functional aspects of hiPSC-CMs, we additionally assessed calcium transient characteristics and membrane potentials. Fluorescent signals of the calcium transients from the hiPSC-CM samples of the control without any stimulation and co-stimulated with an *E* of 5 V/cm and a gradual *E* of 1 to 5 V/cm at an ε of 10% were imaged using fluorescent confocal imaging to investigate how co-stimulation parameters affect the characteristics of calcium transients. For hiPSC-CMs, a time-dependent transitory increase in intracellular calcium ions (Ca^2+^) plays a important role in the contraction. This change in the Ca^2+^ concentration is prompted by the membrane potential that spreads throughout the heart. Hence, detecting the spatiotemporal dynamics of Ca^2+^ is vital to confirming the maturation of a cardiac tissue construct. Hence, we employed a Ca^2+^-sensitive dye as an indicator to confirm intracellular calcium signaling. The calcium transient analysis can complement the ICC and gene expression analyses. Previous studies have suggested that an increase in the amplitude of the Ca^2+^ influx and a decrease in the peak width denote the differentiation of hiPSCs to CMs and the maturation of hiPSC-CMs [30,32,46]. The fluorescent confocal images of the calcium transients indicate that all the hiPSC-CMs have excellent intracellular calcium signaling functions (Fig. 4A). However, it is also evident from the images that hiPSC-CMs were better aligned with simultaneous electro-mechanical co-stimulation (Fig. 4A, II, and B, III) compared to the control sample (Fig. 4A, I). For quantitative analysis, the amplitude, SlopeMax2Pk value in the y-axis (which is the absolute maximum slope from peak start to peak maximum) and full width at half-maximum (FWHM) values were analyzed using the recorded calcium transients for the control and stimulated samples (Movies S2 to S7) and are shown in Fig. 4B. The amplitude values for the control and co-stimulated hiPSC-CMs clearly indicate that the simultaneous electro-mechanical co-stimulation modulated the amplitude of the signals more substantially compared to that of the control sample. The SlopeMax2Pk data obtained indicated a importantly higher SlopeMax2Pk value for the co-stimulated samples when the *E* value was gradually increased from 1 to 5 V/cm at a fixed ε of 10%, compared to the control sample. As shown in Fig. 4B, it is evident from the analyzed results that the hiPSC-CMs co-stimulated with a gradual *E* of 1 to 5 V/cm at a constant 10% of ε showed the narrowest FWHM value. This indicates enhanced maturation characteristics of hiPSC-CMs compared to the control sample or the sample co-stimulated with an *E* of 5 V/cm at an ε of 10%. The obtained results are consistent with results reported previously [30,32,46]. The matured hiPSC-CMs are characterized by increased values of the signal amplitude and SlopeMax2Pk and reduced FWHM values compared to unmatured hiPSC-CMs. Overall, our analyzed results of the gene expression and calcium transients indicate that the maturation of hiPSC-CMs was more prominent when an electrical stimulation with an *E* of 5 and 1 to 5 V/cm was applied at an ε of 10%.

Alterations in the electrical potential across the membrane of hiPSC-CMs also have marked implications in various physiological parameters such as cell signaling and the contraction of cardiac muscle fibers. The analysis of electrical potential and conduction in hiPSC-CMs is important since signal propagation symbolizes a healthy cardiac construct [32,47]. Therefore, the membrane potential was measured to determine how co-stimulation can affect the maturation of the differentiated hiPSC-CMs. We conducted voltage imaging of hiPSC-CMs using a membrane-sensitive fluorescent dye and confocal microscopy. Confocal fluorescent images were obtained from the control without any stimulation and hiPSC-CMs co-stimulated with an *E* of 5 V/cm and 1 to 5 V/cm at a fixed ε of 10%. The fluorescent images revealed excellent electrical membrane functions from all the hiPSC-CMs (Fig. 4C). The results demonstrate that hiPSCs were effectively differentiated into CMs, exhibiting a consistent electrical function related to membrane potential, regardless of the presence or absence of stimulation.

The beating characteristics of hiPSC-CMs usually undergo alterations during their maturation process. Initially, hiPSC-CMs display an immature phenotype, with irregular beating patterns and slower contraction speed when compared to adult cardiomyocytes. Nevertheless, as these cells mature over time in culture, they undergo structural and functional modifications leading to an elevation in their contraction speed. Through our experiments, we were able to confirm this tendency. In Fig. S20, it can be observed that the average contraction speed of the control group, which underwent Wnt signaling modulation, was 3.08 μm/s. In contrast, the groups subjected to electrical stimulations of a constant 5 V/cm and a gradual 1 to 5 V/cm along with mechanical stimulation of 10% strain exhibited progressively increasing average contraction speeds from the control group (3.08 μm/s) to 4.45 and 4.78 μm/s, respectively. The representative beating waveforms and videos for each group of hiPSC-CMs used in the graph of Fig. S20 were shown in Figs. S21 to S23 and Movie S7 to S15, respectively. These results align with the statistical data presented in Fig. 4B, which includes measurements of amplitude, absolute maximum slope from peak start to peak maximum (slopemax2Pk), and FWHM obtained from the calcium transients of hiPSC-CMs. Hence, it can be inferred that there is a correlation between the electrical characteristics of hiPSC-CMs and their beating characteristics. Maturation involves enhancements in ion channels, augmentation of contractile machinery, activation of developmental signaling pathways, and other factors, all of which contribute to the observed changes in contraction speeds during maturation.

Throughout the work, we employed hiPSC-CMs to showcase the effectiveness of the proposed co-stimulation system based on the SMEA device. In addressing the challenge of high mortality due to myocardial infarction [46], there has been substantial research into cardiac-muscle-cell-based treatments. However, these treatments still face notable limitations, including the loss of characteristics found in intact adult heart tissues [47,48]. To overcome these limitations, the differentiation of SCs into CMs, including embryonic SCs [10,48–50] and hiPSCs [9–11,42,51], has been investigated extensively over the last decade. The use of hiPSC-CMs for cardiac tissue engineering is particularly promising because there are no ethical issues. However, hiPSC-CMs have maturation issues [28–30,32,33] because they resemble fetal-state CMs and have not gone through the process of maturation with years of mechanical and electrical stimulations like the human heart after birth. Therefore, the development of approaches to induce their maturation is essential for applications involving hiPSC-CMs. Previous studies on the simultaneous application of multiple stimuli to hiPSC-CMs have reported synergistic effects and a higher level of maturation [52]. However, the detailed maturation mechanism with multiple external stimuli has not been identified, and conclusive outcomes are still lacking under different stimulation conditions. Furthermore, previous research on co-stimulation systems had limits in the hardware for long-term culture with stimulation and constraints. For example, they provided cyclic mechanical stretching stimulation, but a completely integrated electrical and mechanical co-stimulation system could not be established because structurally stable and durable electrodes were difficult to form monolithically on a stretchable substrate [15,53,54]. Alternatively, mechanically static stimulation rather than dynamic stimulation is often used even though electrical stimulation is sufficiently applied [3,7,9,16]. There is a dearth of co-stimulation approaches as a result of the limitations of many technologies.

In developing an in vitro co-stimulation platform which can overcome important issues, we prioritized the fabrication of highly durable electrodes for long-term co-stimulation in culture media. The approach could be realized by creating a highly durable SMEA device with a culture well as a key component. This device facilitated the electro-mechanical co-stimulation of hiPSC-CMs, offering the ability to independently control 2 stimulation parameters while ensuring robust durability. The SMEA device was also transparent, allowing the optical microscopy of cells without damaging the device or cells. The integration of multiple components and multiple functions of the SMEA allowed a simple and compact platform and a small footprint. This advantage allowed the cells to be kept outside of a large incubator during co-stimulation. Additionally, the excellent durability of the device against electrical and mechanical stimulations in culture media allows long-term culture and autoclaving for reuse. Using the developed platform, various electro-mechanical co-stimulations were applied to hiPSC-CMs in search of optimal conditions for their maturation. The shape and arrangement of CM fibers and their maturity level could be manipulated by controlling co-stimulation parameters. Maturity was confirmed through ICC and gene expression analyses. Additionally, analysis of calcium transients, membrane potential, and contraction speed measurements showed that the electrical properties of co-stimulated hiPSC-CMs were improved. The simultaneous application of 10% cyclic mechanical deformations and gradually increasing electrical field resulted in hiPSC-CMs with highly desirable electrical properties.

## Conclusion

Our electro-mechanical co-stimulation system demonstrated the ability to perform electro-mechanical co-stimulation with independent control of electrical and mechanical stimuli without marked damage to the cells or electrodes. This system has the potential to be a valuable tool in the field of regenerative medicine for electrogenic cells including CMs, skeletal muscle cells, and neuronal cells, specifically in the use of hiPSCs. By integrating the SMEA device with stimulation control systems, a wider range of experimental investigations can be conducted beyond the scope of this study. In addition, mechanical cues associated with scaffolds and chemical cues can be added without difficulty. This approach has promising potential for tissue engineering of electrogenic cells, drug screening, toxicity evaluation, and disease modeling using hiPSC-derived cells.

## Ethical Approval

Not applicable.

## Data Availability

The additional data that support the findings of this study are available from the corresponding author upon request.
