# Supplementary material for: Highly Durable, Stretchable Multielectrode Array for Electro-mechanical Co-stimulation of Cells
Source: Biomater Res. 2024 Jun 30;28:0030. doi: 10.34133/bmr.0030 (PMC11214829; doi:10.34133/bmr.0030)
Supplement: Supplementary 1 — Figs. S1 to S23 Movies S1 to S15 [file bmr.0030.f1.zip › biomaterials research_revised SI with clean version.docx]

**Supplementary Information**

**Highly durable, stretchable multielectrode array for electro-mechanical co-stimulation of cells**

A Ri Kim^1^, Sajal Shrivastava^2^, Han-Byeol Lee^3,§^ and Nae-Eung Lee^3,4,5*^

*^1^Department of Nano Science and Technology, Sungkyunkwan University, Suwon, Gyeonggi-do 16419, Republic of Korea*

*^2^Department of Radiology, University of Pittsburgh, Pittsburgh 15213, PA, USA*

*^3^Advanced Institute of Nano Technology, Sungkyunkwan University, Suwon, Gyeonggi-do 16419, Republic of Korea*

*^4^School of Advanced Materials Science & Engineering, Sungkyunkwan University, Suwon, Gyeonggi-do 16419, Republic of Korea*

*^5^Samsung Advanced Institute for Health Sciences & Technology, Sungkyunkwan University, Suwon, Gyeonggi-do 16419, Republic of Korea*

*^§^Currently at Advanced Flash Technology Team, Samsung Electronics Co., Hwasung 18448, Republic of Korea*

**Correspondence:* [*nelee@skku.edu*](mailto:nelee@skku.edu)


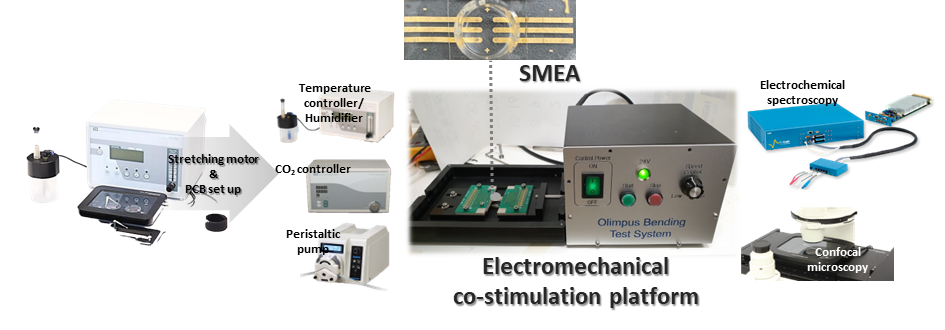


**Fig. S1 Components of electro-mechanical co-stimulation platform.** Modified mini-incubator system houses the SMEA device, PCB for electrical connection, a motor controller for cyclic stretching, and a culture well. The mini-incubator has the ability to control the CO_2_ supply, temperature, and humidity. Peristaltic pumps are used to deliver culture medium to the culture well. This system is connected to an electrochemical potentiostat for the measurement of impedance spectra. The SMEA device can be loaded onto a fluorescence confocal microscope for immunocytochemistry analysis and an optical microscope for cell observations.


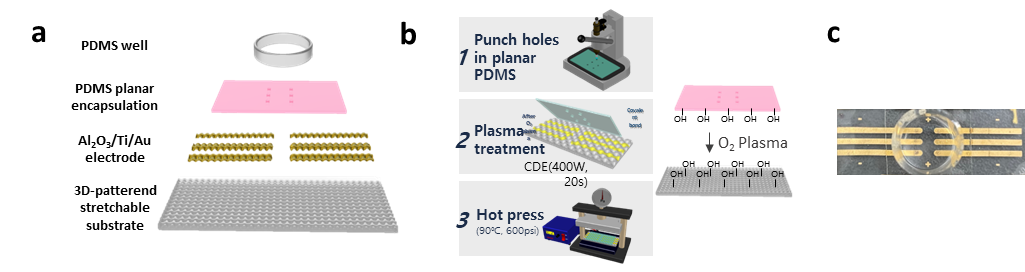


**Fig. S2 Fabrication of electro-mechanical co-stimulation platform. a,** An exploded schematic view of the SMEA device with a PDMS well. **b**, Encapsulation of the Au electrodes with an encapsulation layer. **c**, Top-view photograph of a fabricated SMEA.


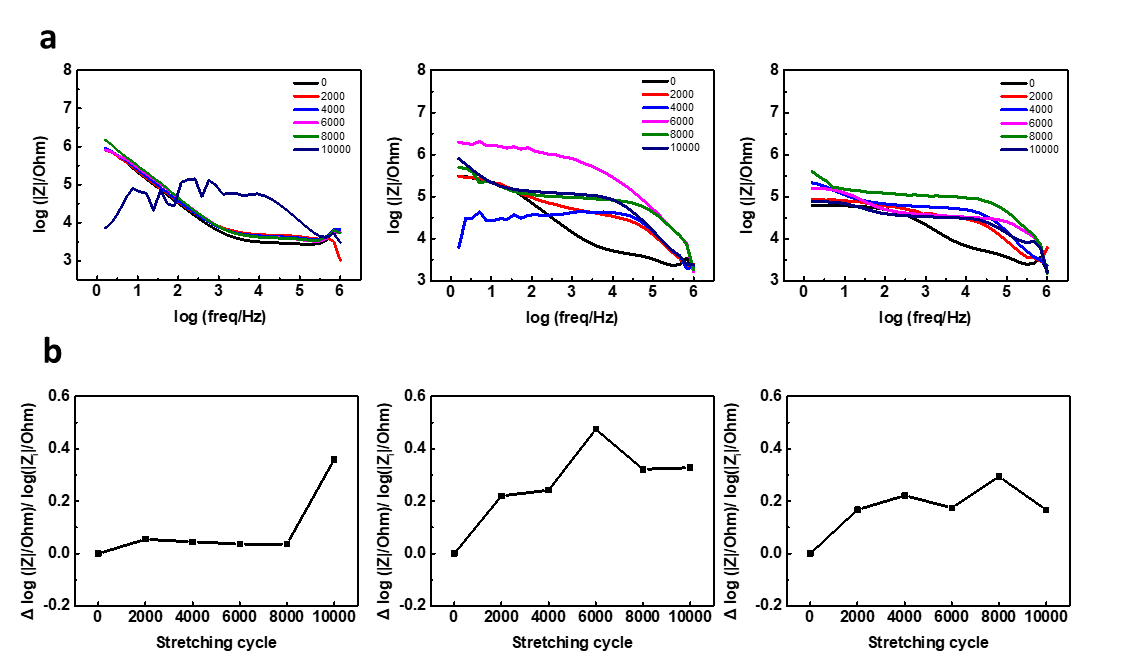


**Fig. S3 Durability evaluation of three SMEA devices on planar substrate under mechanical stimulation.** Impedance spectra **(a)** and the impedance change ratio **(b)** as the number of cyclic stretching increases up to 10,000 times at 10% ε for the device on a planar substrate with the number of stretching cycles varied. The Δlog (|Z|/Ohm)/log (|Z_i_|/Ohm) values were obtained by averaging the impedance values at 10,000 Hz of frequency measured from three electrodes.


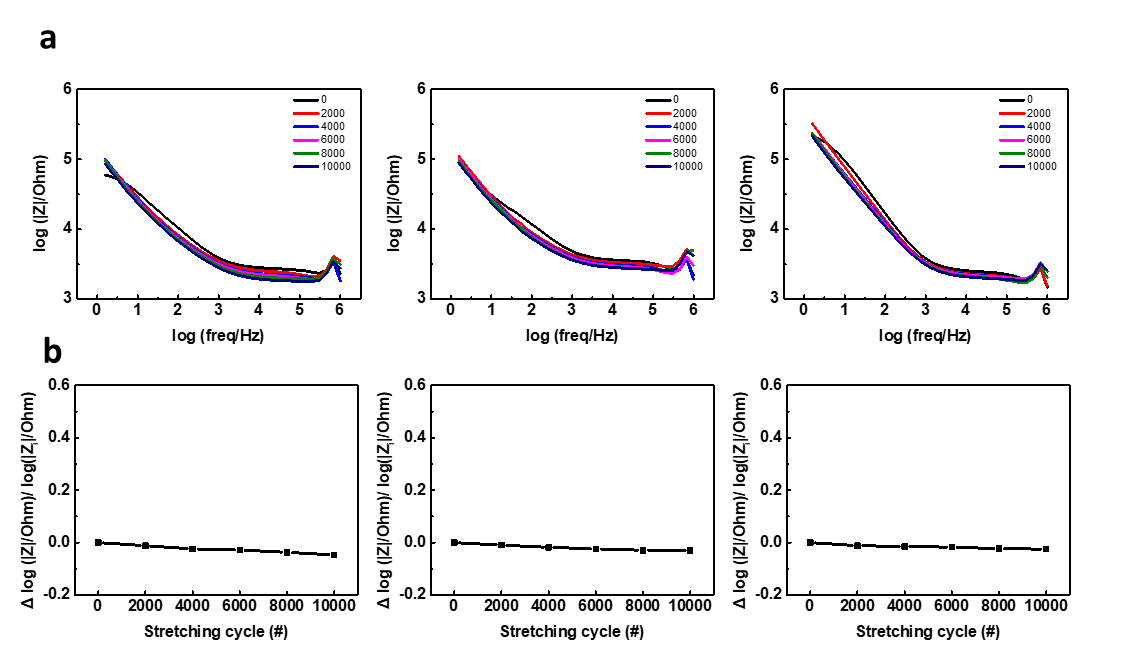


**Fig. S4 Durability evaluation of three SMEA devices on 3D-micropatterned substrate under mechanical stimulation.** Impedance spectra **(a)** and the impedance change ratio **(b)** as the number of cyclic stretching increases up to 10,00 times at 10% ε for the device on 3D-micropatterned substrate. The Δlog (|Z|/Ohm)/log (|Z_i_|/Ohm) values were obtained by averaging the impedance values at 10,000 Hz of frequency measured from three electrodes.


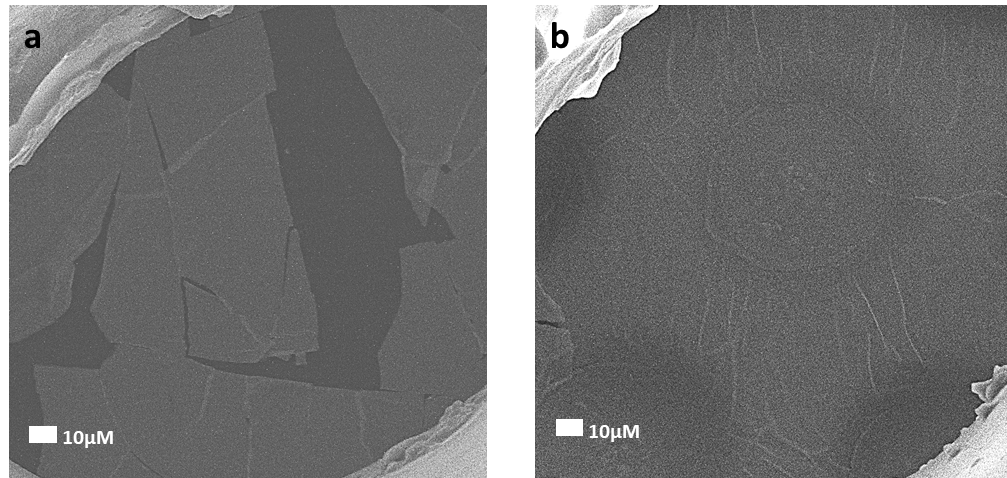


**Fig. S5** FE-SEM images of the Au working electrodes on SMEAs made of planar (a) and 3D-micropatterned (b) substrates after the mechanical cyclic stretching experiment with the varied stretching cycles.


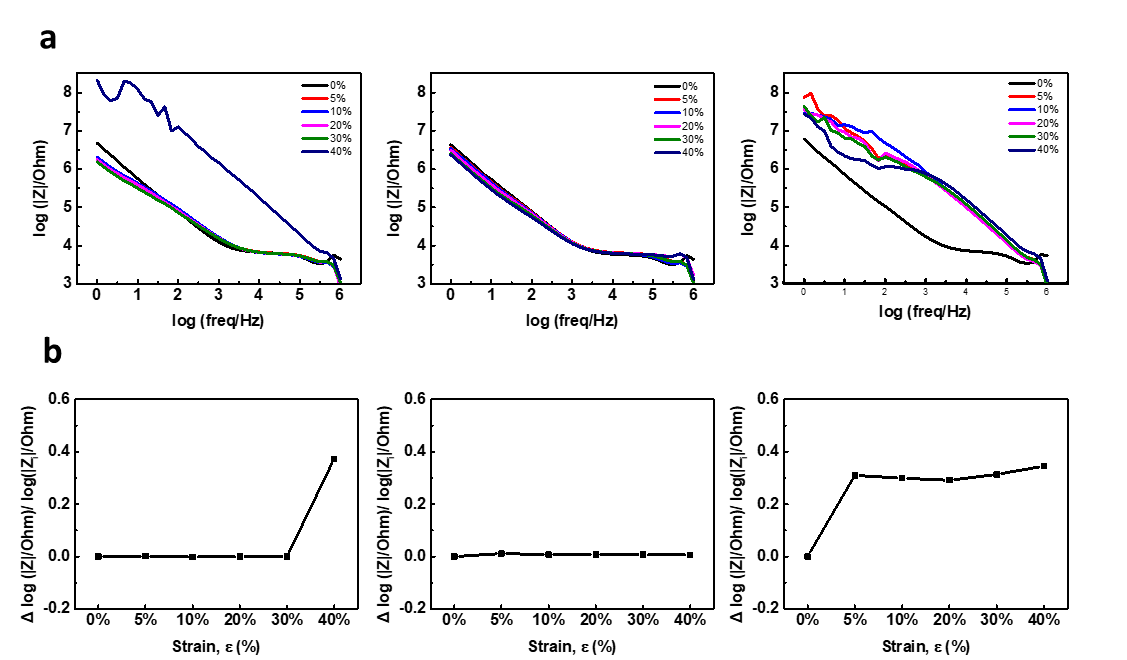


**Fig. S6 Durability evaluation of three SMEA devices on planar substrate under mechanical stimulation. (a)** Impedance spectra and **(b)** impedance change ratio as the stretching ε increases up to 40% ε at the stretching cycles of 10,000 times for the device on the planar substrate. The Δlog (|Z|/Ohm)/log (|Z_i_|/Ohm) values were obtained by averaging the impedance values at 10,000 Hz of frequency measured from three electrodes.


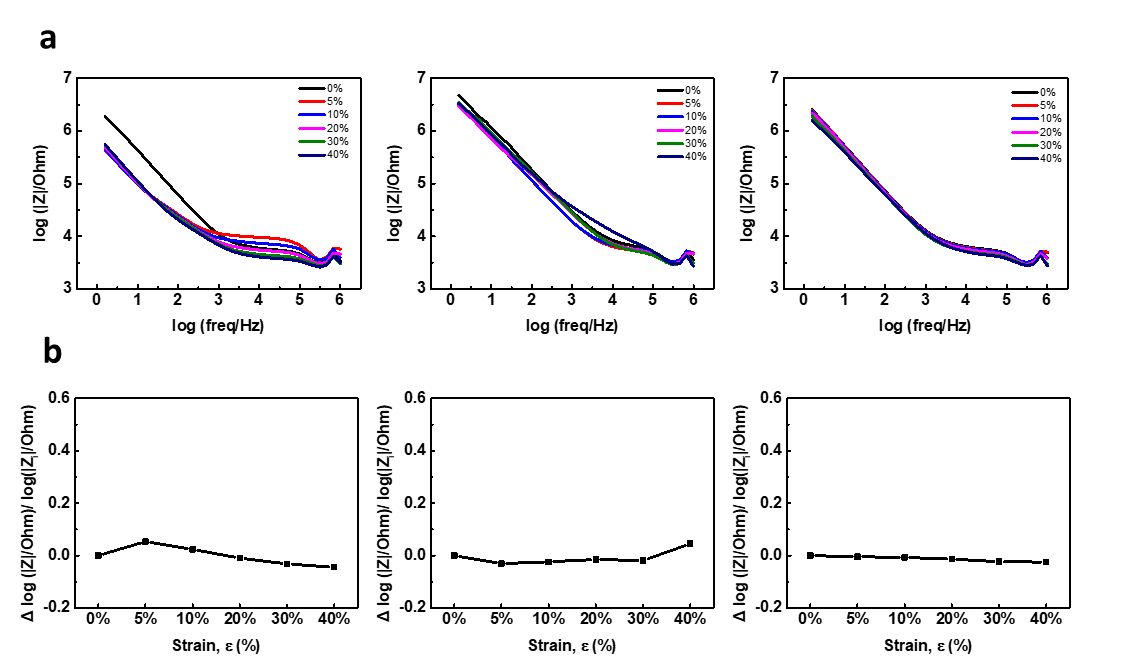


**Fig. S7 Durability evaluation of three SMEA devices on 3D-micropatterned substrates under mechanical stimulation.** The impedance spectra **(a)** and the impedance change ratio **(b)** as the stretching ε increased up to 40% at 10,000 stretching cycles for the device on the 3D-micropatterned substrate. The Δlog (|Z|/Ohm)/log (|Z_i_|/Ohm) values were obtained by averaging the impedance values at 10,000 Hz of frequency measured from three electrodes.


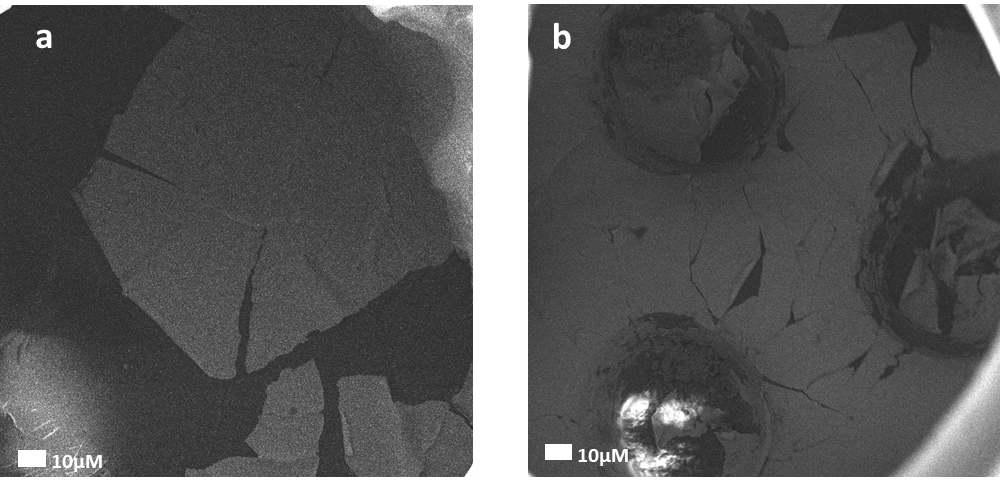


**Fig. S8** FE-SEM images of the Au working electrodes on SMEAs made of planar (a) and 3D- micropatterned (b) substrates after the cyclic stretching experiment with variations in ε.


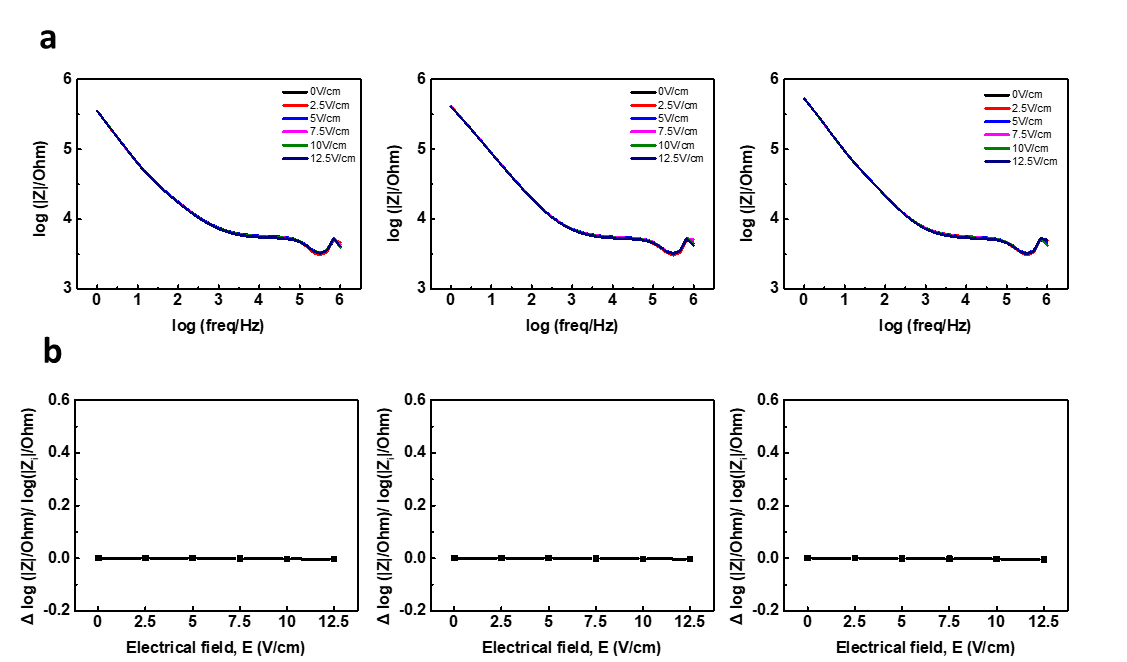


**Fig. S9 Durability evaluation of three SMEA devices on 3D-micropatterned substrate under electrical stimulation.** The impedance spectra **(a)** and the impedance change ratio **(b)** as the biphasic electrical field increases up to 5V for the SMEAs on the 3D-micropatterned substrate. The Δlog (|Z|/Ohm)/log (|Z_i_|/Ohm) values were obtained by averaging the impedance values at 10,000 Hz of frequency measured from three electrodes.


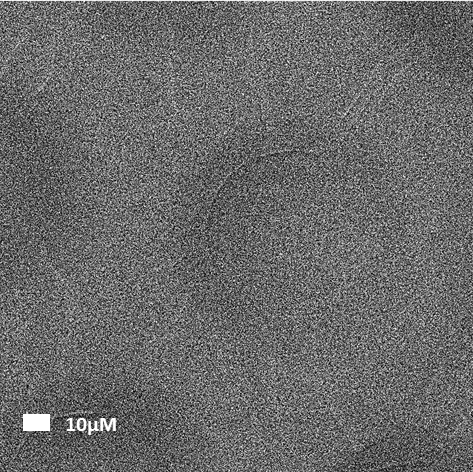


**Fig. S10** FE-SEM image of the Au working electrode on SMEAs made of a 3D-micropatterned substrate after applying the electrical field.


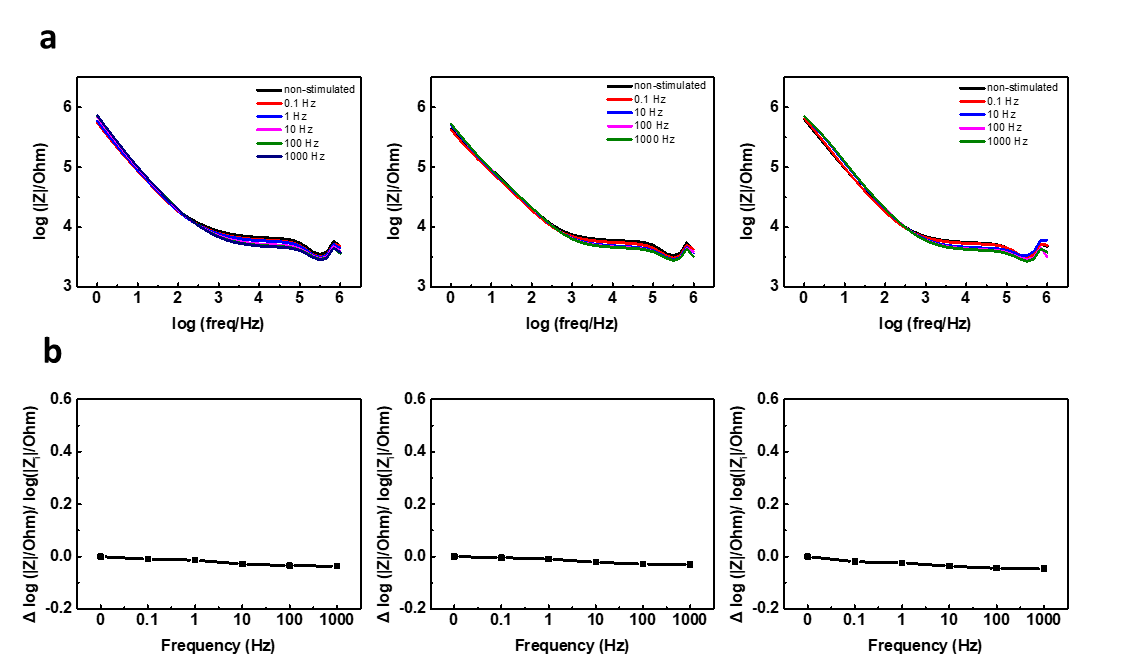


**Fig. S11 Durability evaluation of three MEA devices on 3D-micropatterned substrate under electrical stimulation.** The impedance spectra (upper panels) and the impedance change ratio (lower panels) as the frequency of biphasic electrical stimulation (1 V/cm) increased up to 1,000 Hz for the SMEAs on the 3D-micropatterned substrate. The Δlog (|Z|/Ohm)/log (|Z_i_|/Ohm) values were obtained by averaging the impedance values at 10,000 Hz of frequency measured from three electrodes.


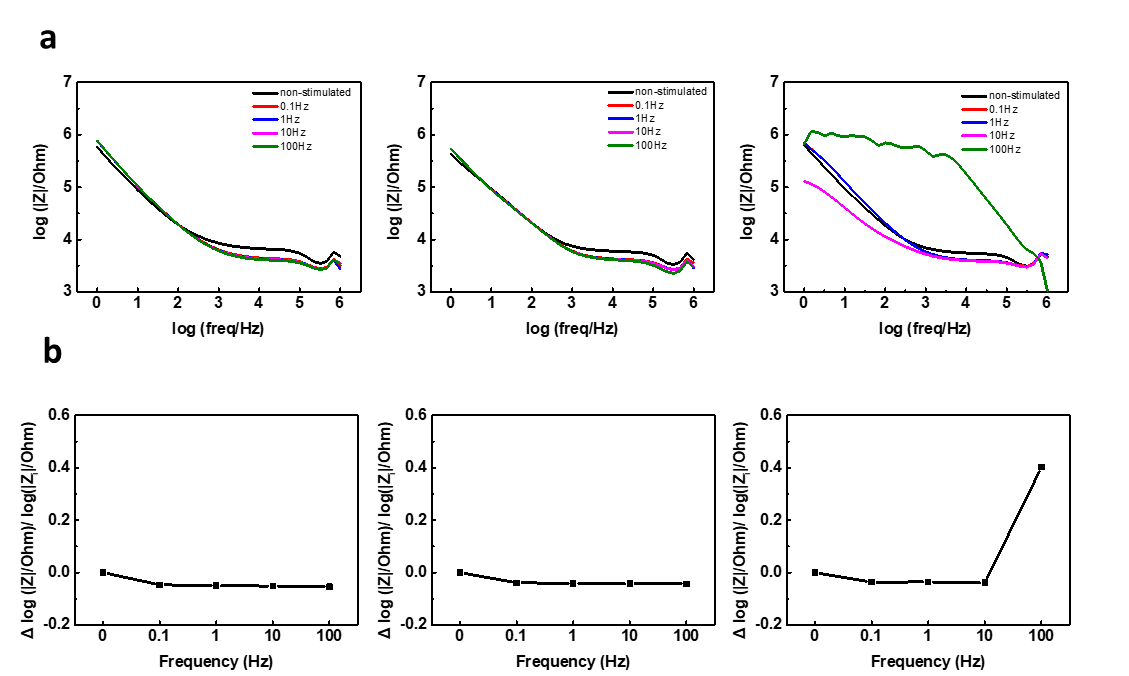


**Fig. S12 Durability evaluation of three SMEA devices on 3D-micropatterned substrate under electrical stimulation.** The impedance spectra **(a)** and the impedance change ratio **(b)** as the frequency of biphasic electrical stimulation (5 V/cm) increased up to 1,000 Hz for the SMEAs on the 3D micropatterned substrate. The Δlog (|Z|/Ohm)/log (|Z_i_|/Ohm) values were obtained by averaging the impedance values at 10,000 Hz of frequency measured from three electrodes.


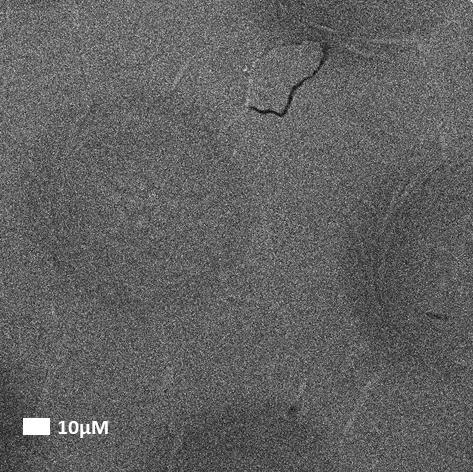


**Fig. S13** FE-SEM image of the Au working electrode on SMEAs after electrical stimulation with the frequency varied.

**Fig. S14 Stability of SMEA with co-stimulation.** Mechanical stimulation of 50,000 cyclic stretching cycles at 10% ε and electrical stimulation of 5 V/cm in cell culture medium were applied. No significant change in the impedance before and after electro-mechanical co-stimulation even when the device was cyclically stretched by 50,000 times at the ε of 10% with the frequency of 1 Hz while the biphasic electrical pulsing with 5 V/cm between two electrodes at a frequency of 1 Hz was applied in culture medium.


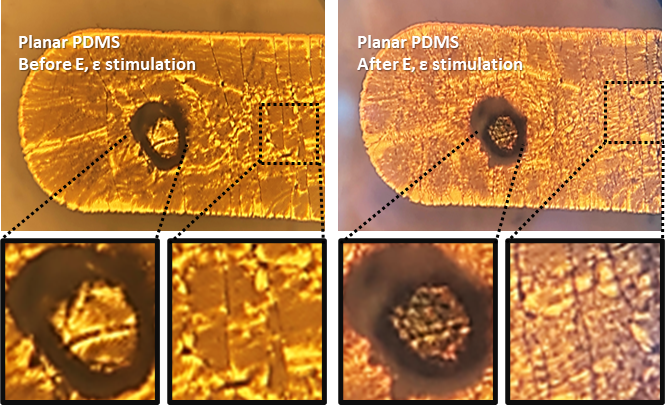


**Fig. S15 Optical images of cyclically stretched SMEAs.** The SMEA was cyclically stretched with 50,000 cycles at 10% ε and electrical pulsing at the amplitude of 5 V/cm in a cell culture medium.


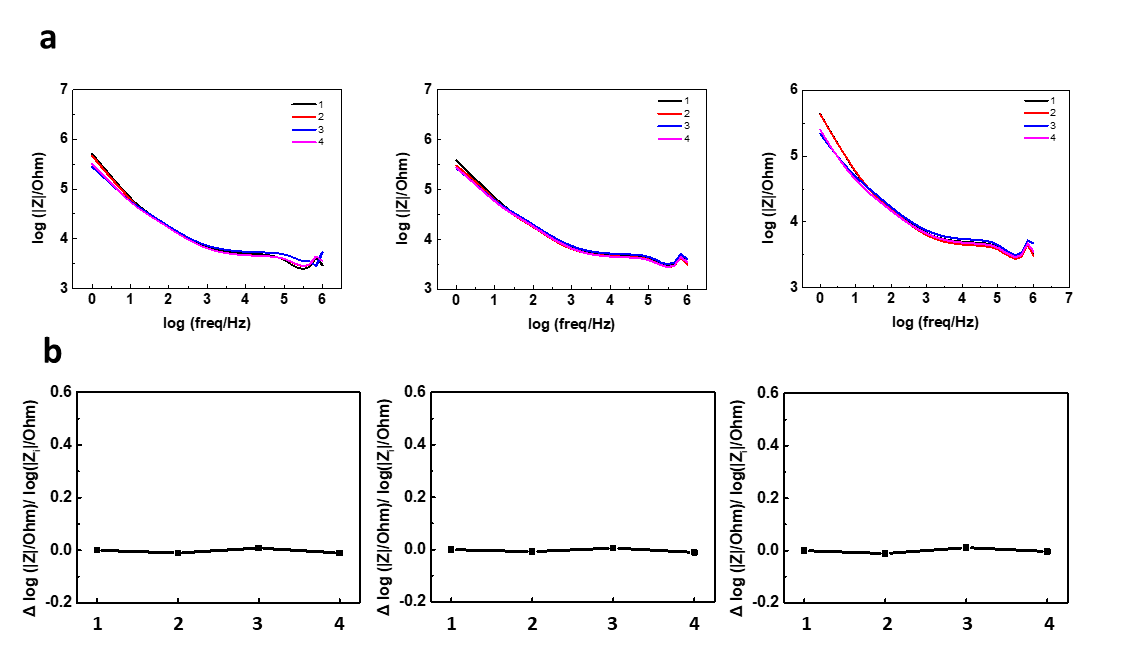


**Fig. S16 Impedance measurement involving co-stimulation and autoclaving.** Before and after autoclaving, the devices underwent 10% ε mechanical stimulation at 1Hz, and 5V/cm biphasic electrical stimulation at 1Hz. Co-stimulations were repeated two times. The averaged Δlog|Z|/log|Z_i_| values at 10,000 Hz obtained from three electrodes in the SMEA were plotted. 1: Before the 1st stimulation, 2: After the 1st stimulation, 3: After autoclaving, 4: After the 2nd stimulation.


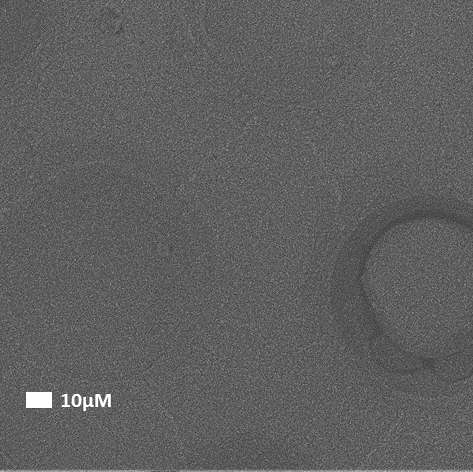


**Fig. S17** FE-SEM image after co-stimulation and autoclaving. The scale bar is 10 μm in all FE-SEM figures. All the Δlog|Z|/log|Z_i_| values were obtained from three working electrodes on SMEAs (n=3).**
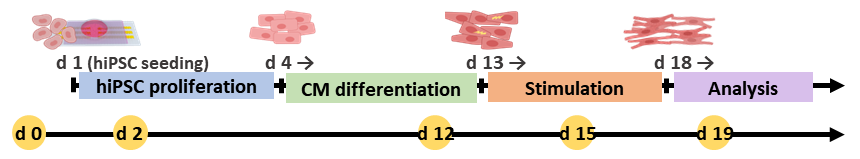
**

**Fig. S18 Experimental flow on the proliferation and differentiation of hiPSCs and the co-stimulation of hiPSC-CMs.** d 1 : hiPSC seeding, d 1 – d 3: proliferation of hiPSCs, d 4 - d 12: differentiation of hiPSCs into CMs, d 13 - d17 : co-simulation, d18 : analysis.


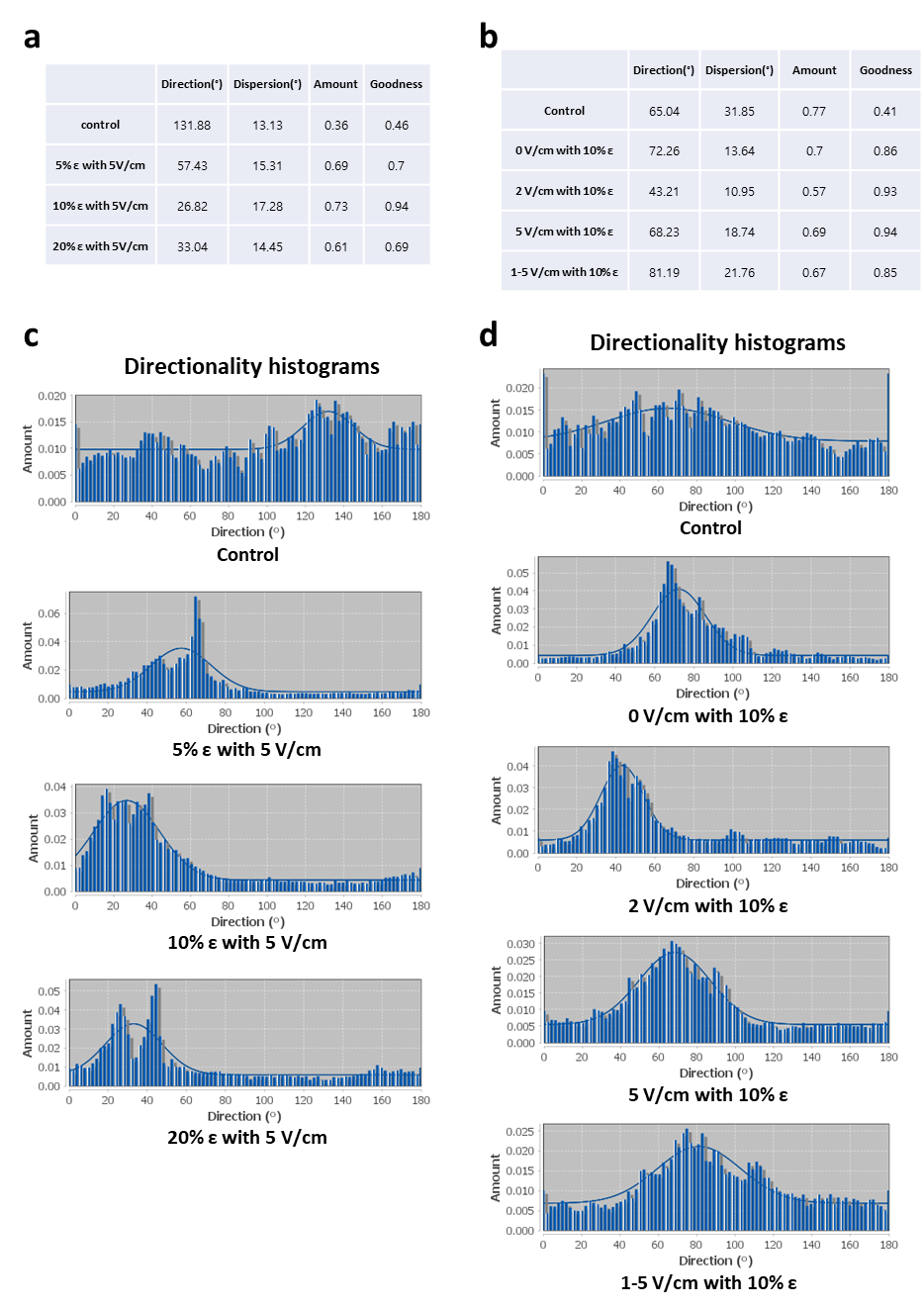


**Fig. S19 Orientation analysis of co-stimulated hiPSC-CMs.** The directionality results and directionality histograms of hiPSC-CMs were obtained from the ICC image in Figure 4a and 4b using ImageJ. **(a)** Characteristics of cell direction from TNNT2 of Figure 4a. **(b)** Characteristics of cell direction from TNNT2 of Figure 4b. **(c)** Directionality histograms from TNNT2 of Figure 4a. **(d)** Directionality histograms from TNNT2 of Figure 4b.

**@ ε = 10%**

**Fig. S20 Analysis of spontaneous beating of the control and co-stimulated hiPSC-CMs.** a, Statistical analysis data on the average contraction speeds obtained from the spontaneous beating of hiPSC-CMs. For control group; the number of samples = 8, the number of videos = 44, number of the peaks = 557. For 5 V/cm group, the number of samples = 7, the number of videos = 64, number of peaks = 972. For 1-5 V/cm group, the number of samples = 12, the number of videos = 64, number of peaks = 934. **: p < 0.01, ****: p < 0.0001 from one-way ANOVA with post hoc Tukey tests. The mechanical strain (ε) was fixed at 10% during the co-stimulation.

**Fig. S21 The representative beating contraction waveform of hiPSC-CM in the control group.**


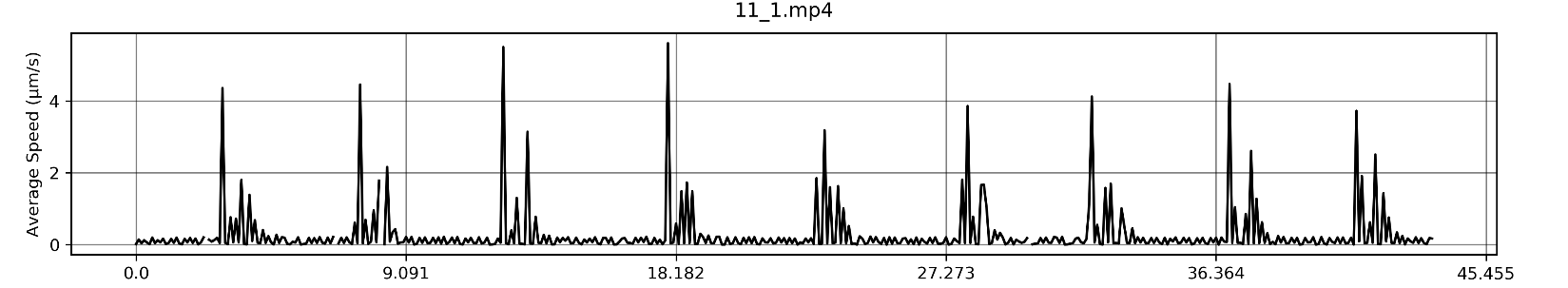

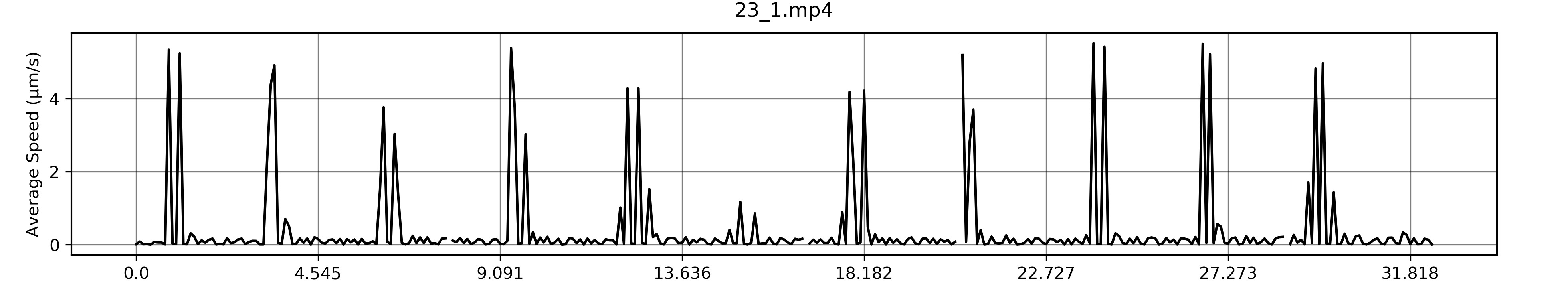

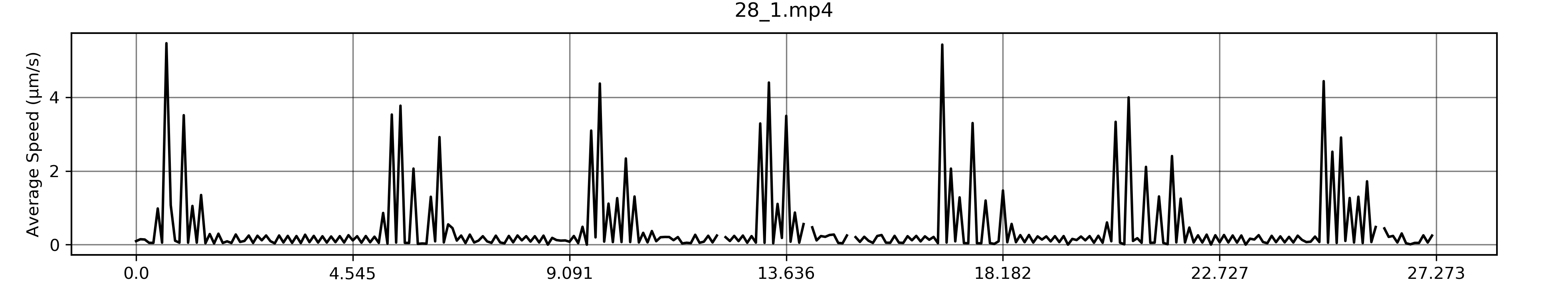


**Fig. S22 The representative beating contraction waveform of hiPSC-CM in the 5 V/cm (E) group with the mechanical stimulation(ɛ) of 10%.**


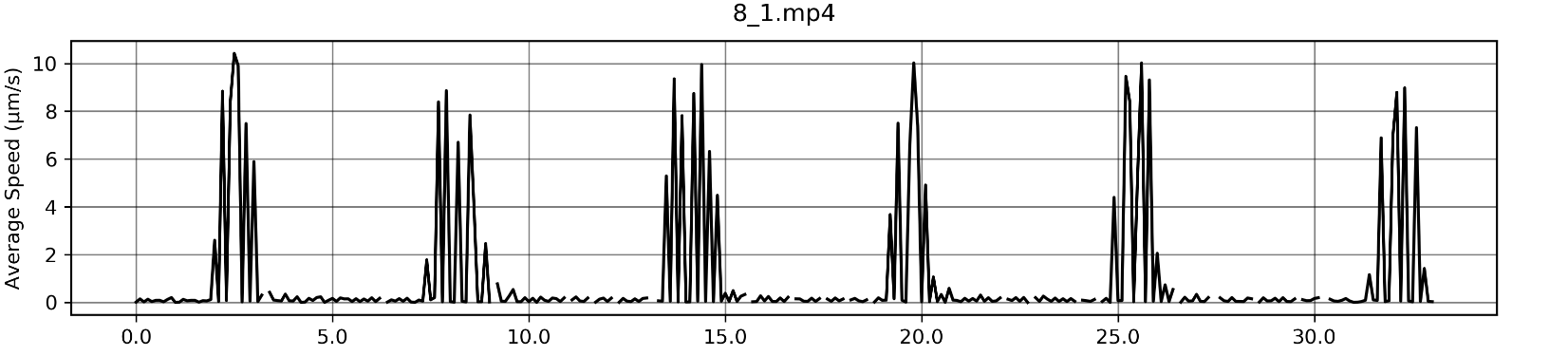

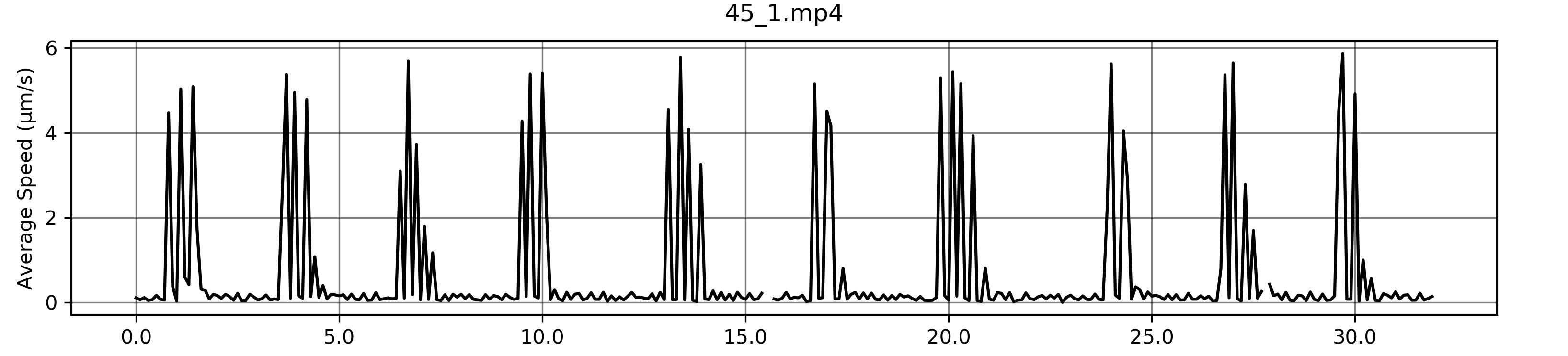

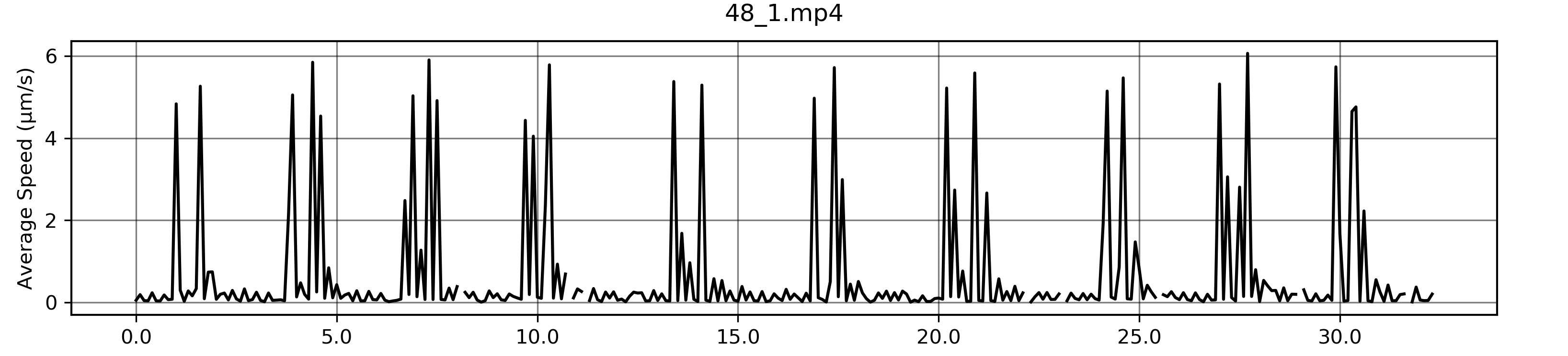


**Supplementary Video 1. Operation of multi-functional tissue engineering system with electrical and mechanical co-stimulation.** The video demonstrates how the contact electrodes of a PCB and SMA are secured within the system, which allows stable electrical and mechanical co-stimulations under uniaxial cyclic stretching. The mechanical stimulation of cyclic stretching is achieved by using a motor attached to the right side of the system.

**Fig. S23 The representative beating contraction waveform of hiPSC-CM in the 1-5 V/cm (E) group with the mechanical stimulation(ɛ) of 10%.**


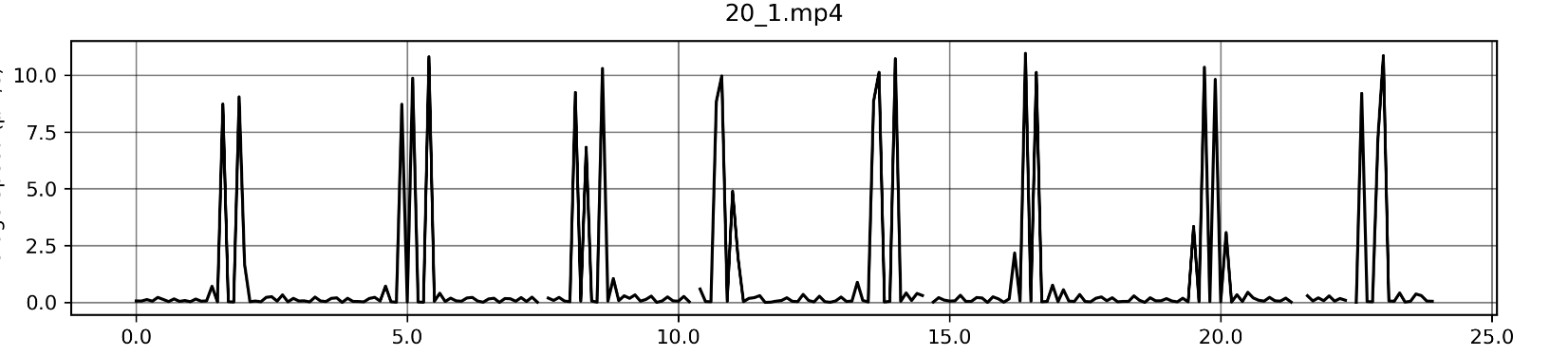

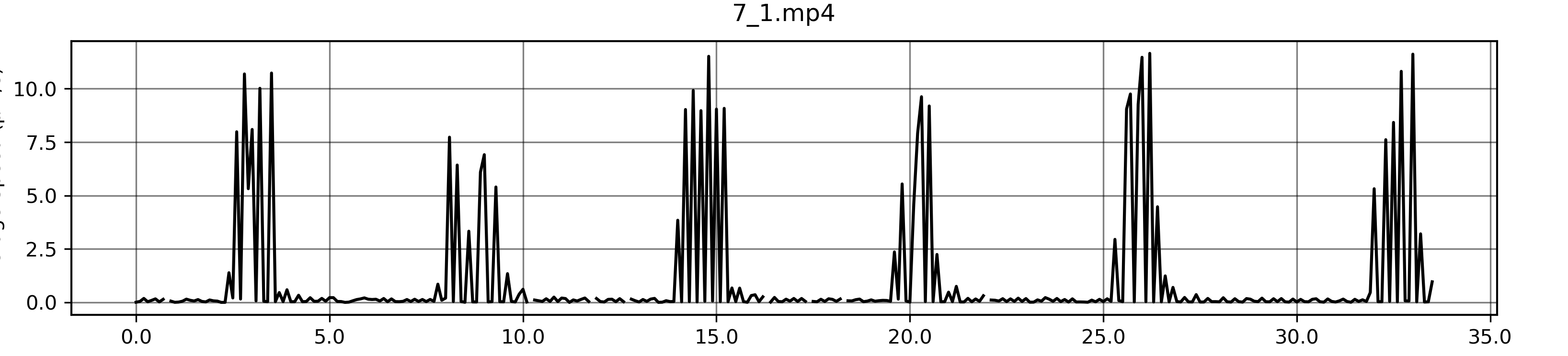

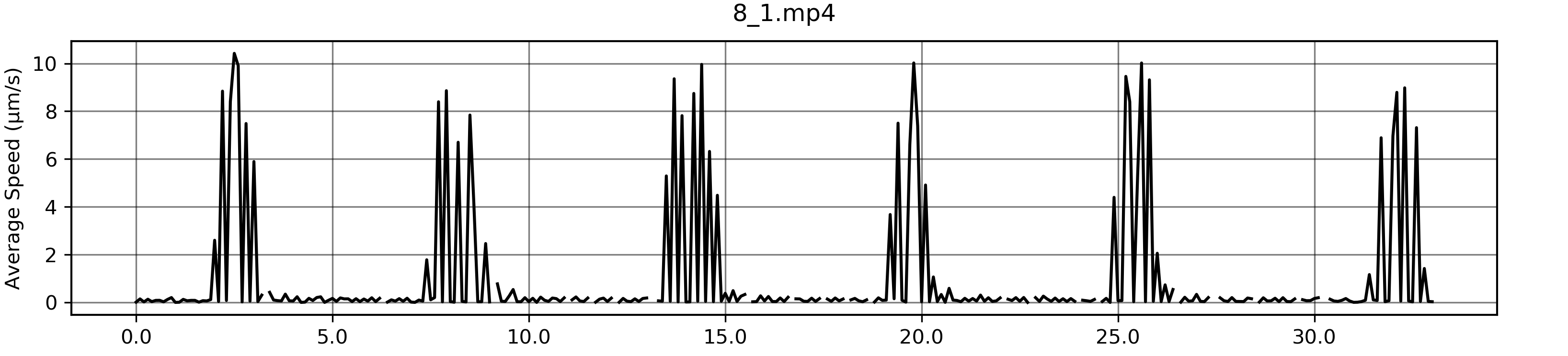


**Supplementary Videos 2 - 6. Recorded videos of calcium transients used for quantitative analysis of the data shown in Fig. 5b, 5c and 5d.** Supplementary videos 1 and 2 feature the control sample, while supplementary videos 3 and 4 show the recordings of the calcium transients from the sample co-stimulated with an E of 5 V/cm at an ε of 10%. Supplementary videos 5 and 6 illustrate the recordings of the calcium transients from the sample co-stimulated with an E of 1-5 V/cm at an ε of 10 %.

**Supplementary Videos 7 – 15 The representative beating video of hiPSC-CMs** **used for quantitative analysis of the data shown in Fig. S21, S22, and S23.** Supplementary videos 7, 8, and 9 feature the control sample, while supplementary videos 10, 11, and 12 show the recordings of the beating from the sample co-stimulated with an E of 5 V/cm at an ε of 10%. Supplementary videos 13, 14, and 15 illustrate the recordings of the beating from the sample co-stimulated with an E of 1-5 V/cm at an ε of 10 %.
